# RIMKLA promotes hypertension by activating PKM2 to trigger VSMC phenotype switch

**DOI:** 10.1016/j.mmr.2026.100056

**Published:** 2026-07-24

**Authors:** Rui Xiang, Wen-Jun Liu, Xin-Rui Zhang, Han Yan, Xin Li, Song Hou, Cheng-Qing Hu, Yun-Tao Hu, Ru-Feng Ma, Jing Li, Yu-Jing Chi, Qing-Hua Cui, Zhen-Zhen Chen, Bin Geng, Ji-Chun Yang

**Affiliations:** a Department of Physiology and Pathophysiology, School of Basic Medical Sciences, State Key Laboratory of Vascular Homeostasis and Remodeling, Center for Non-coding RNA Medicine, Peking University Health Science Center, Beijing 100191, China; b Department of Pathophysiology, School of Basic Medical Science and Public Health, Jiangsu Key Laboratory of Medical Science and Laboratory Medicine, Jiangsu University, Zhenjiang 212013, Jiangsu, China; c Department of Physiology, School of Basic Medical Science, Kunming Medical University, Kunming 650500, China; d Department of Endocrinology, the Second Affiliated Hospital, School of Medicine, Zhejiang University, Hangzhou 310058, China; e Department of Obstetrics and Gynecology, Peking University Third Hospital/National Clinical Research Center for Obstetrics and Gynecology, State Key Laboratory of Female Fertility Promotion, Beijing 100191, China; f Department of Endocrinology, Beijing Chao-Yang Hospital, Capital Medical University, Beijing 100020, China; g Department of Central Laboratory and Institute of Clinical Molecular Biology, Peking University People’s Hospital, Beijing 100044, China; h Department of Biomedical Informatics, School of Basic Medical Sciences, State Key Laboratory of Vascular Homeostasis and Remodeling, Center for Non-coding RNA Medicine, Peking University Health Science Center, Beijing 100191, China; i Beijing Anzhen Hospital of Capital Medical University and Beijing Institute of Heart Lung and Blood Vessel Diseases, Beijing 100029, China; j State Key Laboratory of Cardiovascular Disease, Fuwai Hospital, CAMS & PUMC, Beijing 102308, China; k Department of Cardiology, Peking University Third Hospital, Beijing 100191, China

**Keywords:** Glucose metabolism, Hypertension, Phenotype switch, Pyruvate kinase M2 (PKM2), ribosomal modification protein rimK-like family member A (RIMKLA), Vascular smooth muscle cells (VSMCs)

## Abstract

**Background:**

Hypertension affects around one billion adults worldwide, with abnormal glucose metabolism and vascular smooth muscle cell (VSMC) phenotype switch playing crucial roles in its pathogenesis. Pyruvate kinase M2 (PKM2) is a key glycolytic enzyme, but its regulation and roles in VSMC phenotype switch and hypertension are unknown. Using the Gene Importance Calculator (GIC) to predict gene essentiality, we identified ribosomal modification protein rimK-like family member A (RIMKLA) as a highly relevant gene and explored its regulatory contributions to hypertension.

**Methods:**

Internal mammary arteries from patients with hypertension and normotension, as well as arteries from angiotensin II (Ang II)-induced hypertensive mice, salt-sensitive hypertensive Dahl/SS rats, and spontaneously hypertensive rats, were analyzed in this study. Adenoviruses and adeno-associated viruses were used for ex vivo and in vivo gene overexpression. VSMC-specific RIMKLA or PKM2 knockout mice were generated using the Cre-Loxp system. Arterial tension was measured by wire myography, and blood pressure was assessed by the tail-cuff method and remote radio-telemetry. Protein-protein interactions were determined by co-immunoprecipitation with mass spectrometry. Non-targeted metabolomics, in vitro phosphorylation, adenosine triphosphate (ATP), reactive oxygen species (ROS), and cytoplasmic calcium assays were performed to identify the signaling axis involved.

**Results:**

RIMKLA expression was increased in the medial layer of the internal mammary arteries of patients with hypertension, as well as in hypertensive rat and mouse arteries. RIMKLA overexpression in the mesenteric arteries of Sprague-Dawley rats significantly increased vessel contractility. VSMC-specific RIMKLA overexpression increased arterial contractility and blood pressure in mice. VSMC-specific RIMKLA deletion attenuated Ang II-induced hypertension in mice. Mechanistically, we identified RIMKLA as a scaffold protein that binds to protein-tyrosine phosphatase 1B (PTP1B) and PKM2. RIMKLA phosphorylated PTP1B at tyrosine (Tyr)66, leading to PKM2 dephosphorylation and activation at Tyr105. Once activated, PKM2 enhanced glucose metabolism, increased ROS production, and boosted ATP secretion, driving VSMC phenotype switch. RIMKLA-induced vasoconstriction and hypertension were reversed by VSMC-specific PKM2 deletion. Additionally, a PKM2 inhibitor reduced arterial contractility and blood pressure.

**Conclusions:**

RIMKLA functioned as a scaffold protein kinase, recruiting both PTP1B and PKM2, and orchestrating PTP1B phosphorylation and its subsequent recruitment to activate PKM2. These findings position RIMKLA as a key regulator of PKM2 activation, promoting VSMC phenotype switch and contributing to hypertension.

## 1 Background

Metabolic reprogramming is a hallmark of vascular smooth muscle cell (VSMC) phenotype switch, a key process in vascular remodeling and hypertension progression [1], [2]. Unlike glucose oxidation, which is typically more efficient, VSMCs primarily rely on glycolysis for their energy production. During the VSMC phenotype switch, glycolytic activity increases while glucose oxidation decreases [3].

Inhibiting [4] or genetically deleting [5] pyruvate kinase M2 (PKM2), the key enzyme involved in the final step of glycolysis, has been shown to suppress VSMC proliferation. Conversely, the upregulation of PKM2, facilitated by heterogeneous nuclear ribonucleoprotein A1-mediated splicing of *pre-PKM* messenger RNA (mRNA), promotes VSMC phenotype switch [6]. PKM2 activity is largely regulated by the dephosphorylation of its tyrosine (Tyr)105 residue [7], a process controlled by protein-tyrosine phosphatase 1B (PTP1B). PTP1B itself becomes active upon phosphorylation at Tyr66 [8]. Understanding how PTP1B-PKM2-mediated energy reprogramming contributes to VSMC function and hypertension development is therefore crucial.

To investigate this, we used the Gene Importance Calculator (GIC), a computational tool that predicts gene essentiality, and identified ribosomal modification protein rimK-like family member A (RIMKLA) as a gene of high relevance [9]. RIMKLA ranked within the top 2% of human genes and the top 25% of mouse genes (**Additional file 1:** Table S1), with notable genetic conservation across species (**Additional file 2:** Fig. S1a). Additionally, genome-wide association studies (GWAS) revealed significant associations between single-nucleotide polymorphisms in the enhancer regions of *RIMKLA* and blood pressure regulation (**Additional file 2:** Fig. S1b). RIMKLA, also recognized as family with sequence similarity 80, member A (FAM80A) or N-acetylaspartyl-glutamate synthetase A/II, is functionally uncharacterized. Our recent study [10] identified RIMKLA as a novel regulator of homocysteine (Hcy), which exerts kinase activity to phosphorylate and activate betaine-homocysteine S-methyltransferase 1 (BHMT1), hence promoting Hcy clearance in hepatocytes. Decreased expression of hepatic RIMKLA blunts BHMT1 activity, leading to hyperhomocysteinemia, a crucial risk factor for hypertension. Therefore, it is reasonable to suggest that RIMKLA may be involved in the pathogenesis of hypertension.

This study aims to elucidate the pathophysiological role and molecular basis of RIMKLA in hypertension. By integrating non-targeted metabolomics, protein-protein interaction assays, and genetic manipulations across multiple hypertensive models, we seek to dissect the signaling cascades orchestrated by RIMKLA. These findings may provide mechanistic insights into vascular dysfunction and propose potential therapeutic targets for hypertension management.

## 2 Methods

### 2.1 Animal acquisition and protocols

C57BL/6 J mice (male, 8–10 weeks old), spontaneously hypertensive rats (SHR), and control Sprague-Dawley (SD) rats (male, 8–10 weeks old) were purchased from the Department of Laboratory Science, Peking University Health Science Center. Salt-sensitive hypertensive Dahl/SS rats and control SS-13^BN^ rats (male, 8–12 weeks old) were purchased from Beijing Vital River Laboratory Animal Technology Co., Ltd. Adeno-associated virus vector 2 (AAV2) with smooth muscle-specific promoter (SM22α) carrying *RIMKLA* gene (AAV-RIMKLA) was designed by Beijing Likeli Biotechnology Co., LtdS (China). Male C57BL/6 J mice (8–10 weeks old) were injected via tail vein with AAV-RIMKLA (5×10^11^ vg/mouse) to generate mice with VSMC-specific overexpression of *RIMKLA* [11]. RIMKLA-Loxp mice (RIMKLA^flox/flox^), with two Loxp sites inserted on both sides of exon1 of the mouse *RIMKLA* gene, were constructed by Viewsolid Biotech (China). RIMKLA^flox/flox^ mice on a C57BL/6 J background were crossed with mice specifically expressing Cre in VSMCs driven by the *Tagln* promoter (Tagln-Cre) to generate mice with VSMC-specific deletion of *RIMKLA* gene (RIMKLA^VSMC−/−^). PKM2-Loxp mice (PKM2^flox/flox^) were generously donated by Professor Juan Feng (Peking University Health Science Center). PKM2^flox/flox^ mice on a C57BL/6 J background were crossed with Tagln-Cre mice to generate mice with VSMC-specific deletion of *PKM2* gene (PKM2^VSMC−/−^). Male PKM2^flox/flox^ and PKM2^VSMC−/−^ mice (8–10 weeks old) were subjected to rescue experiments by tail injection with AAV-green fluorescent protein (AAV-GFP) or AAV-RIMKLA to generate mice with VSMC-specific deletion of *PKM2* gene and simultaneous overexpression of *RIMKLA*. Mice and rats were housed in standard animal laboratories maintained at 24 °C under a 12-hour light-dark cycle. All animal care and experimental protocols complied with the Animal Management Rules of the Ministry of Health of the People’s Republic of China and the Guide for the Care and Use of Laboratory Animals of the Peking University Health Science Center. Animal experiments were approved by the Institutional Animal Care and Use Committee of Peking University Health Science Center (BCJB0037).

### 2.2 Gene Importance Calculator

A computational method named GIC to predict the essentiality (importance) of both protein-coding mRNAs and lncRNAs based on key nucleotide sequence characters was previously developed by our laboratory (http://www.cuilab.cn/gic). For predicting the importance of protein-coding genes, GIC outperformed well-established computational scores and CRISPR/Cas9 scores. The higher the predicted score, the more essential the target gene [9].

### 2.3 Mitochondrial respiratory chain function

The oxygen consumption rate (OCR) and extracellular acidification rate (ECAR) in VSMCs were measured by Seahorse XFe24 Extracellular Flux Analyzer (Agilent Technologies, California, USA) following the descriptions of the mitochondrial oxidative phosphorylation (OXPHOS) assay kits (ALS22012, Alicelligent Technologies, Beijing, China). Briefly, the culture medium was replaced with assay medium (ALS22111, Alicelligent Technologies, Beijing, China) supplemented with 10 mmol/L glucose, 1 mmol/L pyruvate, and 2 mmol/L glutamine, adjusted to pH 7.4. Cells were then incubated in a non-CO_2_ incubator at 37 °C for 1 h prior to measurement. During the assay, oligomycin (Oligo; 1.5 μmol/L), carbonylcyanide-4-(trifluoromethoxy)-phenylhydrazone (FCCP; 1.0 μmol/L), and rotenone plus antimycin A (Rot-AA; 1.0 μmol/L) solutions were sequentially injected into the wells of the culture microplate for OCR analysis. Glucose (10 mmol/L), Oligo (1.5 μmol/L), and 2-deoxy-D-glucose (2-DG; 50 m mol/L) solutions were sequentially injected for ECAR analysis. Cells were normalized using an Intelligent Cell Imaging and Analysis System (Falcon S400, Alicelligent Technologies, Beijing, China), and data were evaluated by Wave 2.6.3 software.

### 2.4 Mitochondrial membrane potential assay

After washing with phosphate buffer saline (PBS), VSMCs were treated with 100 nmol/L tetramethylrhodamine methyl ester (TMRM; #M20036, Invitrogen, USA) for 30 min. Then cells were harvested and resuspended in 300 μl PBS (1×10^6^ cells) for flow cytometry (Becton Dickinson, USA) to detect fluorescence intensity reflecting mitochondrial membrane potential.

### 2.5 Adenosine triphosphate measurement

Cultured cells were lysed in adenosine triphosphate (ATP)-Lite Assay Kit lysis buffer (Vigorous Biotechnology Beijing Co., Ltd., China). The culture medium was also collected for ATP determination. For determination of relative ATP level in cells and medium, the ATP content values (nmol) were first normalized to the protein amount (nmol/mg protein) in the same sample, and then normalized to the control values.

### 2.6 Reactive oxygen species assay

After washing with PBS, VSMCs were treated with 20 μmol/L 2’,7’-dichlorodihydrofluorescein diacetate (DCFH-DA; Invitrogen, USA) for 30 min. Cells were then harvested and resuspended in 300 μl PBS (1×10^6^ cells) for flow cytometry (Becton Dickinson, USA) to detect fluorescence intensity reflecting reactive oxygen species (ROS) content.

### 2.7 Molecular-docking

To analyze the interaction affinities between RIMKLA and PKM2, as well as between RIMKLA and PTP1B, a protein docking program named ZDOCK (http://zdock.umassmed.edu/) was used to identify the docking sites [10], [12]. The AlphaFold prediction of RIMKLA (AF-Q8IXN7-F1) was used as the initial models for protein docking, and the Protein Data Bank (PDB) formats of PKM2 (6JFB) and PTP1B (1A5Y) structural domains were downloaded from the PDB database (http://www.rcsb.org/). The protein docking figures were prepared in PyMOL (https://pymol.org/2).

### 2.8 In vitro phosphorylation assay

PKM2 or PTP1B protein from rat primary VSMCs was immunoprecipitated using protein A/G Sepharose beads crosslinked with PKM2 or PTP1B antibody. The obtained protein (20 μg) was incubated with recombinant RIMKLA (rRIMKLA; 20 ng/ml; #P05051, Solarbio, China) in kinase buffer (25 mmol/L HEPES, 1 mmol/L DTT, 50 mmol/L NaCl, 2 mmol/L EGTA, 5 mmol/L MgSO_4_) containing 50 μmol/L ATP. The reaction mixtures were incubated at 37 °C for 2 h, followed by immunoblotting. The protocol was adapted from previous studies [13], [14].

### 2.9 Co-immunoprecipitation and glutathione S-transferase pull-down

A Pierce immunoprecipitation (IP) kit (#26147, Thermo Fisher Scientific, USA) was used for IP assays. VSMCs were treated with Ad-RIMKLA (with His tag) to overexpress *RIMKLA* in cells; IP experiments were then performed using anti-His antibodies, with IgG as a negative control. The obtained IP proteins were analyzed by SDS-PAGE, followed by silver nitrate staining. The positive staining bands were then subjected to mass spectrometry (MS) sequencing analysis. PKM2 with a glutathione S-transferase (GST) tag fusion protein (PKM2-GST; #P2262, Beyotime, China) was expressed *in vitro* and combined with glutathione Sepharose 4B resin (#P2020, Solarbio, China). The 4B resin with PKM2-GST was then added to cell lysate containing the target protein RIMKLA and incubated at 4 °C overnight. 4B resin with Null-GST served as a negative control.

Other detailed methods are provided in **Additional file 2: Methods**, and detailed materials and resources used in this study are presented in **Additional file 2:** Table S1 and **Additional file 1:** Table S2**.**

### 2.10 Statistical analysis

All data were analyzed with ImageJ v1.53e and GraphPad Prism v10.4.1, and are presented as mean±standard error of the mean (SEM). The normal distribution of data was assessed by Shapiro-Wilk test. Student’s *t*-test or one/two-way ANOVA followed by Bonferroni post hoc analysis was used to compare normally distributed data. Within-test multiple comparison corrections were applied using Tukey’s multiple comparisons test. Statistical significance of differences for non-normally distributed data was analyzed by Mann-Whitney *U* test (two groups), or Kruskal-Wallis test (multiple groups) followed by Dunn’s post hoc analysis. *P*<0.05 was considered statistically significant. For *P*-values less than 0.001, they are denoted as *P*<0.001; for *P*-values greater than or equal to 0.001, the *P*-values are reported to three decimal places.

## 3 Results

### 3.1 RIMKLA upregulation in the arterial VSMCs of patients and animal models with hypertension

**Fig. 1:**
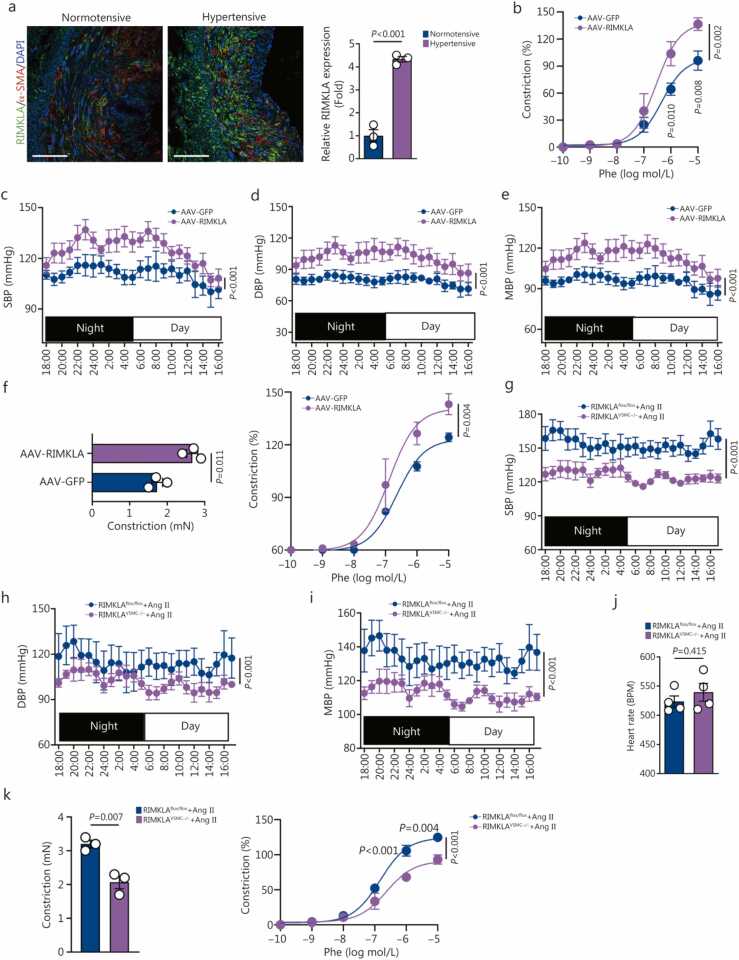
VSMC RIMKLA promotes arterial contraction and heightens blood pressure. a Representative images of immunofluorescent staining of RIMKLA protein in internal mammary artery of hypertensive patients and normotensive controls and the quantitative data analysis (n=3). Scale bar=75 μm. b Mesenteric arteries of SD rats preincubated with Ad-RIMKLA have stronger contractive response to Phe than those of control arteries treated with Ad-GFP (n=5). VSMC-specific overexpression of RIMKLA (AAV-RIMKLA) in C57BL/6 J mice increased SBP (c), DBP (d) and MBP (e) levels when compared to control mice (AAV-GFP) (n=5). f Thoracic arteries of AAV-RIMKLA-injected mice had stronger constriction in response to potassium (left) and Phe (right) than those of control mice injected with AAV-GFP (n=3). RIMKLA^VSMC−/−^ mice had lower SBP (g), DBP (h) and MBP (i) levels than control RIMKLA^flox/flox^ mice after Ang II treatment for 2 weeks (n=4). j RIMKLA^VSMC−/−^ mice had comparable heart rate to RIMKLA^flox/flox^ mice after Ang II treatment for 2 weeks (n=4). k Thoracic arteries of RIMKLA^VSMC−/−^ mice had a weaker contraction response to potassium (left) and Phe (right) than those of RIMKLA^flox/flox^ mice after Ang II treatment (n=3). Arteries were preincubated with 100 nmol/L Ang II for 10 min before contractility was determined. P-value for (a, f-left, j, k-left) are analyzed by unpaired t-test. Others are determined by two-way ANOVA followed by Bonferroni correction and within-test multiple corrections using Tukey’s multiple analysis. RIMKLA. Ribosomal modification protein rimK-like family member A; DAPI. 4,6’-diamidino-2-phenylindole; α-SMA. α-smooth muscle actin; Ad-RIMKLA. Adenoviral RIMKLA; GFP. Green fluorescent protein; Ang II. Angiotensin II; SBP. Systolic blood pressure; DBP. Diastolic blood pressure; MBP. Mean blood pressure; Phe. Phenylephrine.

To investigate the involvement of RIMKLA in VSMC phenotype switch and hypertension, we assessed its expression in arterial tissues from humans with hypertension and animal models of hypertension. Human arteries were obtained in a previous study [15], and the clinical characteristics are shown in **Additional file 1:** Table S3. Immunofluorescence analysis revealed significantly elevated RIMKLA protein expression in the VSMCs of internal mammary arteries from patients with hypertension compared with individuals with normotension (Fig. 1**a**). Upregulation of RIMKLA was observed in the thoracic aortas of angiotensin II (Ang II)-induced hypertensive mice (**Additional file 2:** Fig. S2a), the mesenteric and thoracic arteries of salt-sensitive hypertensive Dahl/SS rats (**Additional file 2:** Fig. S2b**, c**), and SHR (**Additional file 2:** Fig. S2d**, e**). These findings suggest a consistent pattern of increased RIMKLA expression in hypertensive conditions, implicating its involvement in disease pathogenesis.

### 3.2 VSMC RIMKLA promotes arterial contraction and increases blood pressure

We determined the alterations in vasoconstriction and relaxation *ex vivo*, as well as blood pressure changes *in vivo,* in the context of *RIMKLA* overexpression or deletion. *RIMKLA* overexpression in the mesenteric arteries of SD rats by pre-incubating with adenoviral RIMKLA (Ad-RIMKLA, with GFP tag) for 12 h (**Additional file 2:** Fig. S3a) increased the contractile response to potassium (**Additional file 2:** Fig. S3b) and phenylephrine (Fig. 1**b**), without interfering with endothelial-dependent (**Additional file 2:** Fig. S3c) and endothelial-independent (**Additional file 2:** Fig. S3d) relaxation. To determine RIMKLA’s effect *in vivo*, AAV-RIMKLA was injected via the tail vein to induce VSMC-specific *RIMKLA* overexpression (**Additional file 2:** Fig. S3e) without changing its expression in the liver, kidney, heart and muscle (**Additional file 2:** Fig. S3f). This increased RIMKLA immunofluorescent staining in the mesenteric artery (**Additional file 2:** Fig. S3g) compared with control mice receiving AAV-GFP injection.

Blood pressure was dynamically (4, 6, 8, and 12 weeks) measured by the tail-cuff method after AAV-GFP or AAV-RIMKLA injection (**Additional file 2:** Fig. S3h**-j**). In AAV-RIMKLA mice, arterial systolic blood pressure (SBP) was continuously higher from 4 to 8 weeks after injection, while mean blood pressure (MBP) was increased from 6 to 12 weeks (**Additional file 2:** Fig. S3h**, j**). Diastolic blood pressure (DBP) was elevated at 8 weeks post-injection (**Additional file 2:** Fig. S3i), with no significant changes in heart rate (**Additional file 2:** Fig. S3k), compared with AAV-GFP-injected mice. To confirm the blood pressure changes, blood pressure was also determined by remote radio-telemetry in conscious freely moving mice using an HD-X10 radio-telemetry probe catheter after 8 weeks of virus injection. Consistently, AAV-RIMKLA-treated mice showed increased SBP, DBP, and MBP (increase of approximately 20 mmHg) (Fig. 1**c-e**), without heart rate changes (**Additional file 2:** Fig. S3l). In keeping with the *in vivo* effects, AAV-RIMKLA-treated mouse thoracic aortas showed increased contraction in response to potassium (Fig. 1**f**, left) and phenylephrine (Fig. 1**f**, right), with no change in acetylcholine (Ach)- and sodium nitroprusside (SNP)-induced dilation (**Additional file 2:** Fig. S3m).

RIMKLA^VSMC−/−^ were generated via RIMKLA^flox/flox^ mice hybridization with Tagln-Cre mice (**Additional file 2:** Fig. S4a**, b**). *RIMKLA* deletion in VSMCs was verified by *Loxp* and *Cre* expression (**Additional file 2:** Fig. S4c), along with diminished *RIMKLA* mRNA (**Additional file 2:** Fig. S4d) and protein levels in the mesenteric artery (**Additional file 2:** Fig. S4e). In physiological conditions, there was no significant difference in blood pressure, heart rate, cardiac morphology, heart weight, or heart to body weight ratio between RIMKLA^VSMC−/−^ mice and RIMKLA^flox/flox^ mice (**Additional file 2:** Fig. S4f**-h**).

Importantly, *RIMKLA* deletion attenuated Ang II-induced hypertension, including the reduction in SBP (Fig. 1**g**) by around 30 mmHg, DBP by around 10 mmHg (Fig. 1**h**), and MBP by around 20 mmHg (Fig. 1**i**), without heart rate changes (Fig. 1**j**). The *ex vivo* experiments showed that the thoracic arteries of RIMKLA^VSMC−/−^ mice had a weaker contraction response than those of RIMKLA^flox/flox^ mice after Ang II treatment (Fig. 1**k**), with no change in relaxation (**Additional file 2:** Fig. S4i). Moreover, neither VSMC-specific deletion nor *RIMKLA* overexpression altered serum NO bioavailability (**Additional file 2:** Fig. S5a).

Hcy and Ang II, two key risk factors for vascular diseases, upregulated RIMKLA protein expression in cultured VSMCs (**Additional file 2:** Fig. S5b). However, *RIMKLA* overexpression in VSMCs did not significantly alter the extracellular or intracellular Hcy concentration, regardless of Hcy treatment (**Additional file 2:** Fig. S5c). These loss-/gain-of-function experiments indicate that RIMKLA is induced by certain risk factors, such as Hcy and Ang II, which target VSMC contractility but not dilation, promoting hypertension.

### 3.3 RIMKLA promotes VSMC phenotype switch

**Fig. 2:**
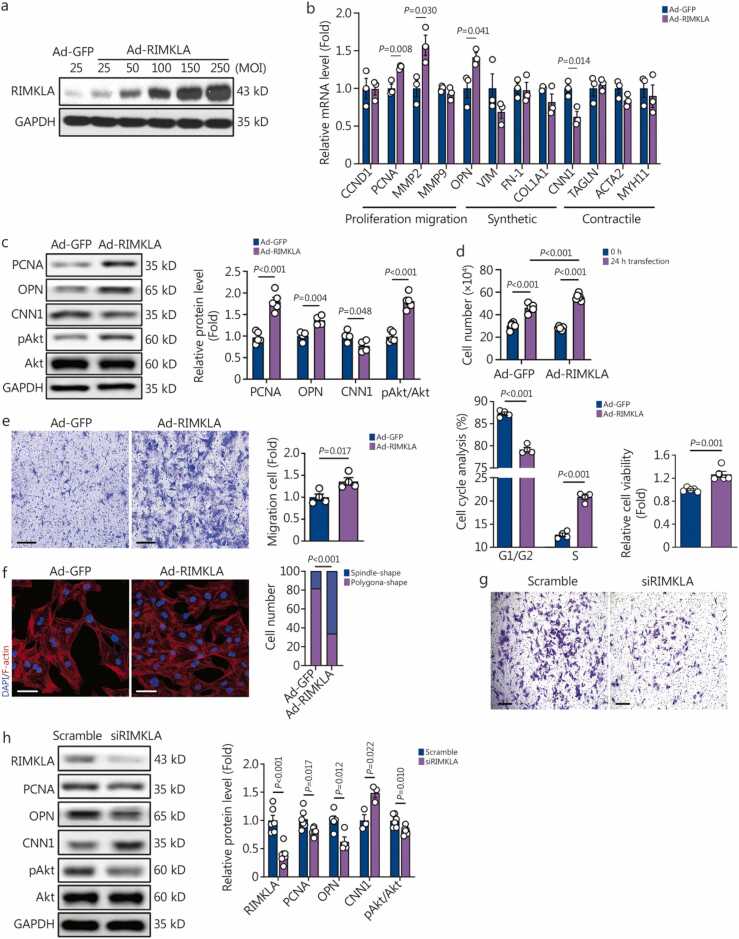
RIMKLA promotes phenotype transition of VSMC from contractile phenotype to proliferative phenotype. a Treatment with adenovirus RIMKLA (Ad-RIMKLA) dose-dependently increased RIMKLA protein level. Primary rat VSMCs were treated with different dose of Ad-RIMKLA for 24 h. Ad-GFP (25 MOI) is used as control. b Overexpression of RIMKLA elicited phenotype switch of VSMCs from contractile phenotype to proliferative phenotype (n=3). Rat primary VSMCs were treated with Ad-GFP or Ad-RIMKLA for 24 h. c Overexpression of RIMKLA elevated PCNA and OPN protein levels, and activated Akt, whereas decreased CNN1 protein level in VSMCs (n=4–5). d Overexpression of RIMKLA promoted proliferation of VSMCs as evaluated by cell number counting, cell cycle assays, and cell viability (n=4–6). e Overexpression of RIMKLA promoted the migration of VSMCs as assessed by Transwell assay (n=4). Scale bar=200 μm. f Representative immunofluorescence images (left) of F-actin (red) stained with phalloidin in primary rat VSMCs that were treated with Ad-GFP or Ad-RIMKLA for 24 h followed by serum starvation for another 24 h. DAPI staining (blue) indicates the nucleus. Scale bar=50 μm. RIMKLA overexpression decreased the percentages of spindle-like (contractile phenotype) and increased polygonal-like (synthetic) VSMCs (right). Data were obtained from 100 cells from three independent experiments. g RIMKLA knockdown restrained the migration of VSMCs as assessed by Transwell assay. Representative images were shown in the panel. Scale bar=100 μm. h siRNA silencing of RIMKLA reduced OPN and PCNA protein levels, and inhibited Akt phosphorylation with enhanced CNN1 protein level (n=3–6). P-value for (f) is determined by χ^2^ test, and others are determined by unpaired t-test. MOI. Multiplicity of infection; CCND1. Cyclin D1; PCNA. Proliferating cell nuclear antigen; MMP. Matrix metalloproteinase; OPN. Osteopontin; VIM. Vimentin; FN-1. Fibronectin; COL1A1. Collagen-1; CNN1. Calponin 1; TAGLN. Transgelin or smooth muscle 22α (SM22α); ACTA2. Alpha-2 smooth muscle actin or α-smooth muscle actin (α-SMA); MYH11. Smooth muscle myosin heavy chain 11; RIMKLA. Ribosomal modification protein rimK-like family member A; DAPI. 4,6’-diamidino-2-phenylindole; Ad-RIMKLA. Adenoviral RIMKLA; GFP. Green fluorescent protein.

The transition of VSMCs from a contractile to a synthetic phenotype plays a decisive role in the pathogenesis of hypertension [2]. Our previous study has identified that protein kinase B (Akt) phosphorylation and activation contribute to VSMC phenotype switch [15]. *RIMKLA* overexpression in VSMCs (Fig. 2**a; Additional file 2:** Fig. S5d) promoted Akt phosphorylation and the synthetic phenotype, as demonstrated by the upregulation of proliferating cell nuclear antigen (*PCNA*) and osteopontin (*OPN*) mRNA and protein expression, along with reduced calponin 1 (*CNN1*) mRNA and protein expressions (Fig. 2**b, c**). *RIMKLA* overexpression also increased cell number, S-phase cell number, cell viability (Fig. 2**d**), and cell migration ability (Fig. 2**e**), but it decreased the characteristic spindle-shaped morphology (Fig. 2**f**) of cultured VSMCs. In the mesenteric arteries of AAV-RIMKLA-treated mice, OPN protein expression was increased, whereas CNN1 expression was reduced (**Additional file 2:** Fig. S6a**, b**). In support, siRNA knockdown of *RIMKLA* reduced Akt phosphorylation and VSMC phenotypic switching (Fig. 2**g, h; Additional file 2:** Fig. S6c**, d**). OPN expression was decreased, while CNN1 expression was increased in the mesenteric artery of RIMKLA^VSMC−/−^ mice (**Additional file 2:** Fig. S6e**, f**) and in the renal artery of Ang II-induced hypertensive RIMKLA^VSMC−/−^ mice (**Additional file 2:** Fig. S6g**, h**). In addition, RIMKLA^VSMC−/−^ mice exhibited amelioration of Ang II-induced artery remodeling, as demonstrated by the reduction in mesenteric medial layer thickness and area (**Additional file 2:** Fig. S7a), with no difference in the renal and thoracic arteries (**Additional file 2:** Fig. S7b**, c**). Meanwhile, VSMC-specific overexpression of *RIMKLA* exhibited little effect on artery remodeling (**Additional file 2:** Fig. S8a**-e**), cardiac systolic function (**Additional file 2:** Fig. S8f**-h**), and cardiac morphology (**Additional file 2:** Fig. S8i). These data suggest that RIMKLA activation is involved in the progression of Ang II-induced hypertension by promoting VSMC phenotype switch.

### 3.4 RIMKLA enhances VSMC ATP synthesis and ROS production

**Fig. 3:**
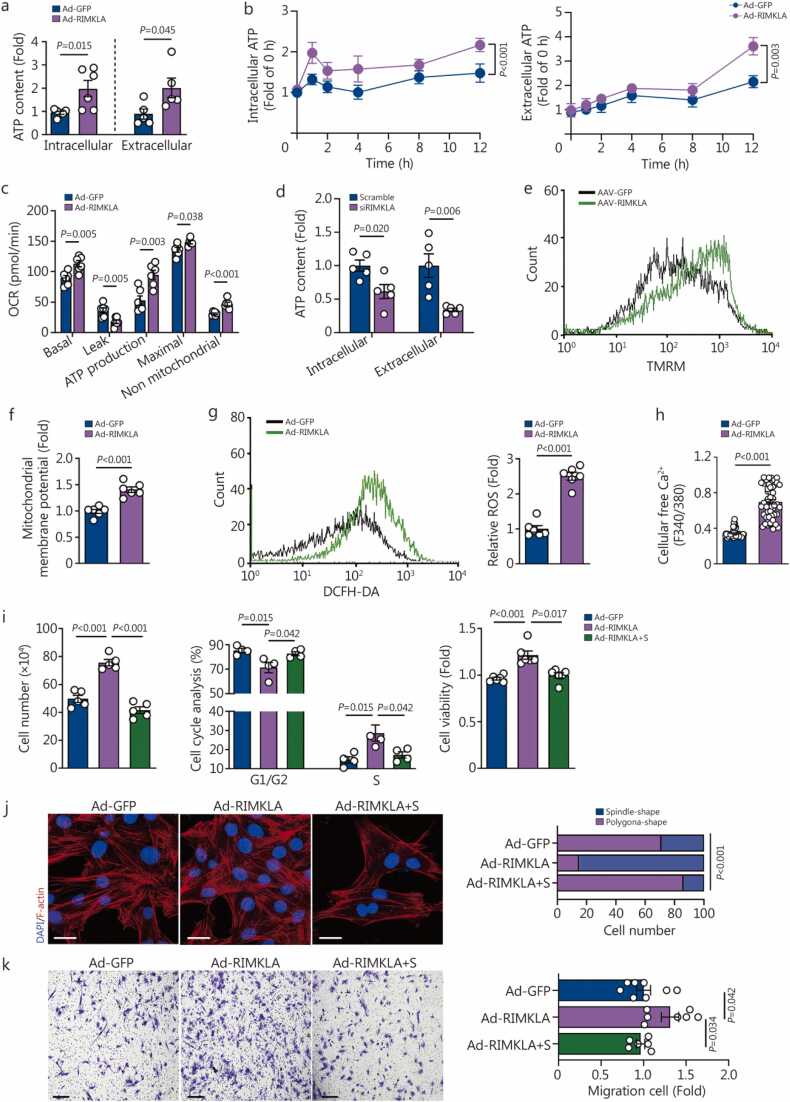
RIMKLA promotes ATP synthesis and ROS production in VSMCs. a Treatment of Ad-RIMKLA increased intracellular and extracellular ATP content (n=5). VSMCs were treated with Ad-GFP or Ad-RIMKLA for 24 h. b Dynamic measurement of intracellular and extracellular ATP content after Ad-RIMKLA infection (n=6). c RIMKLA overexpression increased the oxygen consumption rate (OCR) in VSMCs. Basal: basal respiration; leak: proton leak; maximal: maximal respiration. ATP production was calculated by subtracting proton leak from basal respiration. d siRIMKLA transfection decreased intracellular and extracellular ATP content in VSMCs (n=5). VSMCs were treated with scrambled or siRIMKLA for 24 h. Overexpression of RIMKLA increased mitochondrial membrane potential (e, f) and ROS production (g). Representative images were shown in panels (e) and (g-left), and quantitative data were presented in panels (f) and (g-right) (n=6). h RIMKLA overexpression increased cellular Ca^2+^ levels in VSMCs. Data were obtained from more than 100 cells from 5 independent experiments. i VSMCs were treated with Ad-RIMKLA in the absence or presence of 40 μmol/L suramin (S) for 24 h. Suramin blocked RIMKLA-induced proliferation of VSMC as evaluated by cell number counting, cell cycle, and cell viability assays (n=4–6). j Representative immunofluorescence images of F-actin (red) stained with phalloidin in primary rat VSMCs treated with Ad-GFP or Ad-RIMKLA in the absence or presence of 40 μmol/L suramin for 24 h followed by serum starvation for another 24 h. DAPI staining (blue) indicated the nucleus. Scale bar=50 μm. Quantitative data of the percentages of spindle-like (contractile phenotype) or polygonal-like (synthetic) VSMCs in three groups. Data were obtained from 100 cells from three independent experiments. k Suramin reversed RIMKLA-promoted migration of VSMC as evaluated by transwell assay (n=5–7). Scale bar=100 μm. P-values are analyzed by two‑way ANOVA with Bonferroni and Tukey’s corrections (b), Mann‑Whitney U test (h), one‑way ANOVA with Bonferroni post hoc (i, k), and chi‑square test (j). Others are determined by an unpaired t-test. RIMKLA. Ribosomal modification protein rimK-like family member A; DAPI. 4,6’-diamidino-2-phenylindole; Ad-RIMKLA. Adenoviral RIMKLA; GFP. Green fluorescent protein; ATP. Adenosine triphosphate; ROS. Reactive oxygen species.

ATP synthesis/release and ROS production are critical factors in triggering the VSMC phenotypic switch [15]. We found that *RIMKLA* overexpression resulted in increased intracellular and extracellular ATP concentrations in VSMCs (Fig. 3**a**). A dynamic assessment of ATP concentration showed a significant increase in intracellular ATP within 1 h of *RIMKLA* overexpression, which remained elevated for up to 12 h (Fig. 3**b**, left). Additionally, extracellular ATP concentration was significantly elevated after 12 h of *RIMKLA* overexpression (Fig. 3**b**, right). Mitochondrial respiration analysis further confirmed that RIMKLA enhances the OCR and ECAR (Fig. 3**c; Additional file 2:** Fig. S9a). Conversely, *RIMKLA* knockdown led to a reduction in both ATP synthesis and secretion in VSMCs (Fig. 3**d**). *RIMKLA* overexpression also increased mitochondrial membrane potential (Fig. 3**e, f**), total ROS production (Fig. 3**g**), and intracellular free Ca^2+^ concentration (Fig. 3**h**).

To confirm the roles of ATP and ROS signaling in RIMKLA-mediated VSMC phenotypic switch, ATP-P2 receptor inhibitor (suramin), ROS scavenger [N-acetyl-L-cysteine (NAC)], and xanthine oxidase inhibitor [febuxostat (FB)] were used. First, suramin suppressed RIMKLA-induced VSMC proliferation, as evaluated by cell counting (Fig. 3**i**, left), cell cycle (Fig. 3**i,** middle), cell viability (Fig. 3**i,** right), and cell morphology (Fig. 3**j**) assays. Suramin also inhibited RIMKLA-promoted VSMC migration (Fig. 3**k**) and suppressed RIMKLA-stimulated PCNA expression, Akt activation (**Additional file 2:** Fig. S9b), and intracellular Ca^2+^ elevation **(Additional file 2:** Fig. S9c). Functionally, suramin preincubation restrained RIMKLA-triggered vasoconstriction (**Additional file 2:** Fig. S9d) but not vasodilation (**Additional file 2:** Fig. S9e). The findings suggest that ATP-P2 signaling contributes to RIMKLA-induced VSMC phenotype switch and vasoconstriction.

Xanthine is a metabolic intermediate of ATP degradation to uric acid, and it was decreased by *RIMKLA* overexpression in VSMCs in the metabolomics analysis (**Additional file 2:** Fig. S10a**, b**), with increased xanthine oxidase activity causing uric acid accumulation (**Additional file 2:** Fig. S10c). Suramin inhibited the RIMKLA-induced increase in xanthine oxidase activity and uric acid production (**Additional file 2:** Fig. S10c). Both ROS scavenging by NAC and xanthine oxidase inhibition by FB suppressed RIMKLA-promoted VSMC proliferation (**Additional file 2:** Fig. S10d). Taken together, P2 receptor activation and the subsequent increase in xanthine oxidase activity, uric acid production, and ROS accumulation appear to be involved in RIMKLA-mediated VSMC phenotype switch.

### 3.5 RIMKLA activates PKM2 to regulate VSMC glucose metabolism

**Fig. 4:**
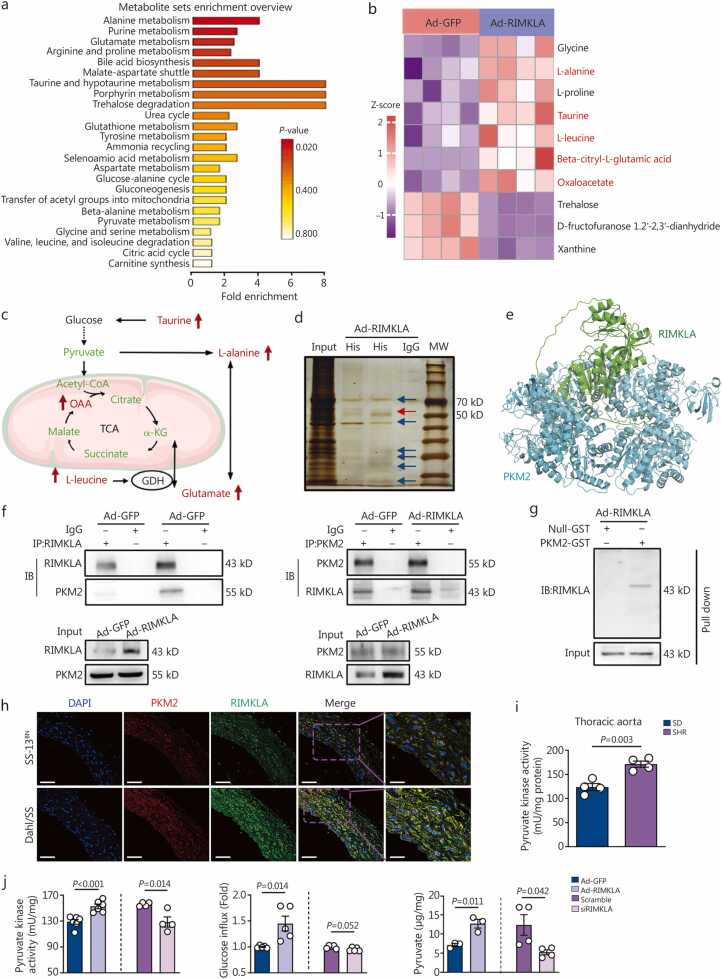
RIMKLA enhances glucose metabolism in VSMCs through activating PKM2. a Metabolite set enrichment analysis of differential metabolites after overexpression of RIMKLA in VSMCs (n=4). b Heat map of 10 differential metabolites with the most significant change was presented (n=4). c Five of seven remarkably upregulated differential metabolites (L-alanine, taurine, L-leucine, beta-citryl-L-glutamic acid, and oxaloacetate) were classified as the key intermediate metabolites involved in glucose metabolism and TCA cycle. d Primary rat VSMCs were treated with Ad-RIMKLA (with His tag) for 24 h prior to immunoprecipitation (IP) (n=3). IP proteins were separated by SDS-PAGE, and then subjected to silver nitrate staining. The potential positive bands (indicated by the arrows) were subjected to mass spectrometry analysis. PKM2 was identified as a target protein interacting with RIMKLA in the band indicated by the red arrow. e ZDOCK program (http://zdock.umassmed.edu) predicted the possible interaction model between RIMKLA and PKM2. f The interaction between RIMKLA and PKM2 was verified by cross IP and immunoblotting (IB) (n=3). Input is the whole protein pre-cleaned with Pierce control agarose resin. g GST-pull down assay indicated the direct interaction between RIMKLA and PKM2 (n=3). h In VSMCs of mesenteric arteries of salt-sensitive hypertensive Dahl/SS rats, PKM2 and RIMKLA were colocalized (n=3). Scale bar=25 μm for the last merged images, and 50 μm for other images. i In the thoracic artery of SHR rats, pyruvate kinase activity was increased (n=4). j RIMKLA overexpression or silencing elevated or repressed pyruvate kinase activity, glucose influx, and pyruvate production in VSMCs (n=3–6). P-values are determined by unpaired t-test. VSMC. Vascular smooth muscle cell; RIMKLA. Ribosomal modification protein rimK-like family member A; Ad-RIMKLA. Adenoviral RIMKLA; GFP. Green fluorescent protein; PKM2. Pyruvate kinase M2; TCA. Tricarboxylic acid; GST. Glutathione S-transferase; MW. Molecular weight; DAPI. 4,6’-diamidino-2-phenylindole.

To further determine the mechanism of RIMKLA-induced ATP synthesis and ROS production in VSMCs, non-targeted metabolomics was performed after *RIMKLA* overexpression. Totally, 172 metabolites were identified and classified into 34 categories. Among them, amino acids were the most abundant metabolites (55 metabolites) (**Additional file 2:** Fig. S11, raw data presented in **Additional file 1:** Tables S4, S5). Metabolite set enrichment analysis revealed that RIMKLA likely regulated alanine metabolism, purine metabolism, and pyruvate metabolism, among others (Fig. 4**a**). The 10 (7 upregulated and 3 downregulated) metabolites (including xanthine) with the most significant changes after *RIMKLA* overexpression are presented in Fig. 4**b**. Further analysis after literature mining [16], [17], [18], [19], [20] revealed that among these 10 metabolites, 5 upregulated metabolites (L-alanine, taurine, L-leucine, beta-citryl-L-glutamic acid, and oxaloacetate) were tightly associated with glucose metabolism and the tricarboxylic acid (TCA) cycle (Fig. 4**c**), suggesting that RIMKLA may regulate glucose metabolism in VSMCs.

To investigate the mechanism by which RIMKLA may modulate glucose metabolism in VSMCs, co-immunoprecipitation (Co-IP) and MS were performed to identify the potential proteins that interacted with RIMKLA. After SDS-PAGE separation of Co-IP products and silver staining, the arrow-indicated bands (Fig. 4**d**) were subjected to MS analysis. Importantly, PKM2, one of the key enzymes controlling glycolysis, was identified as one of the target proteins in the band indicated by the red arrow with matched molecular weight (MS data presented in **Additional file 1:** Table S6). First, ZDOCK [12] molecular docking predicted the potential interaction between RIMKLA and PKM2 (Fig. 4**e**). Furthermore, Co-IP using RIMKLA antibody (Fig. 4**f**, left) or PKM2 antibody (Fig. 4**f**, right), and GST pull-down confirmed the direct RIMKLA-PKM2 interaction (Fig. 4**g**).

RIMKLA-PKM2 colocalization was verified in cultured VSMCs, and it was increased after *RIMKLA* overexpression (**Additional file 2:** Fig. S12a). RIMKLA-PKM2 colocalization was also confirmed in mesenteric artery media and it was increased under hypertensive conditions (Fig. 4**h; Additional file 2:** Fig. S12b). The pyruvate kinase activity in arterial tissues was also increased by about 38% in hypertensive arteries compared with control arteries (Fig. 4**i**). In cultured VSMCs, *RIMKLA* overexpression increased pyruvate kinase activity, glucose influx, and pyruvate production, while *RIMKLA* knockdown reduced all of them (Fig. 4**j**). Taken together, these results demonstrate that RIMKLA may enhance VSMC glycolysis by binding to and activating PKM2.

### 3.6 RIMKLA recruits PTP1B to dephosphorylate and activate PKM2

**Fig. 5:**
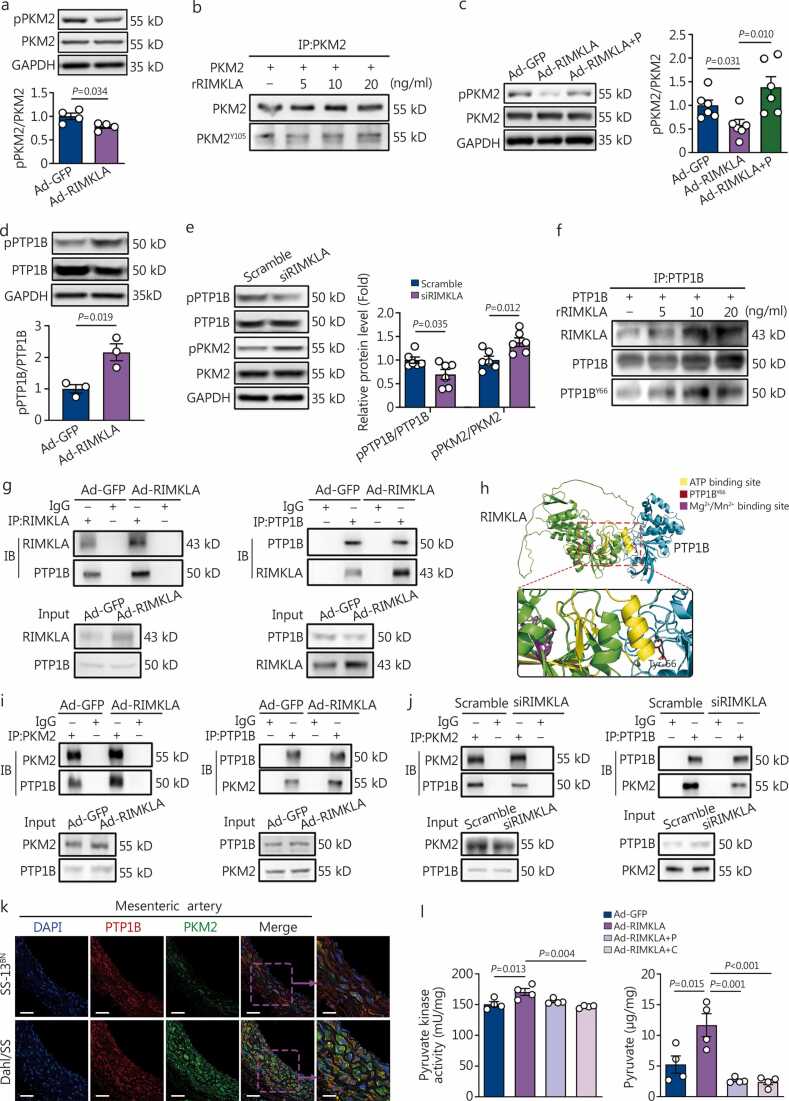
RIMKLA recruits PTP1B to dephosphorylate and activate PKM2 in VSMCs. a Overexpression of RIMKLA reduced PKM2 phosphorylation (Tyr105) (n=4). VSMCs were treated with Ad-GFP or Ad-RIMKLA for 24 h before immunoblotting. b In vitro phosphorylation assay revealed that RIMKLA had little effect on PKM2 phosphorylation (n=3). PKM2 protein (20 µg) was incubated with rRIMKLA protein (5, 10, 20 ng/ml) in kinase buffer for 2 h before immunoblotting assays. c Treatment with PTP1B inhibitor (PTP1B-IN-2, 15 μmol/L) reversed RIMKLA-induced decrease of PKM2 phosphorylation (Tyr105) (n=6). d Overexpression of RIMKLA promoted PTP1B phosphorylation (Tyr66) (n=3). e RIMKLA knockdown reduced PTP1B phosphorylation (Tyr66) with increased PKM2 phosphorylation (Tyr105) (n=6). f In vitro phosphorylation assay revealed that RIMKLA directly phosphorylated PTP1B at Tyr66 (n=4). PTP1B protein (20 μg) was incubated with rRIMKLA protein (5, 10, 20 ng/ml) in kinase buffer for 2 h before immunoblotting assays. g The interaction between RIMKLA and PTP1B was verified by cross IP and IB (n=3). Input is the whole protein pre-cleaned with Pierce control agarose resin. h ZDOCK program predicted the possible interaction model between RIMKLA and PTP1B. Tyr66 of PTP1B (red) was located near the ATP binding site (yellow, the predicted protein kinase domain) of RIMKLA. Overexpression of RIMKLA increased the interaction between PKM2 and PTP1B (i), whereas RIMKLA knockdown decreased the interaction between PKM2 and PTP1B (j) in VSMCs (n=3). k In mesenteric arterial VSMCs of salt-sensitive hypertensive Dahl/SS rats, PKM2 and PTP1B were colocalized (n=3). Scale bar=50 μm or 25 μm. l Treatment with PTP1B inhibitor (PTP1B-IN-2, 15 μmol/L) or PKM2 inhibitor (C3K, 0.5 μmol/L) reversed RIMKLA-promoted increase in pyruvate kinase activity and pyruvate production in VSMCs (n=4). P-values of (a, d, e) are analyzed by unpaired t-test. Others are determined by one-way ANOVA followed by Bonferroni post hoc analysis. VSMC. Vascular smooth muscle cell; RIMKLA. Ribosomal modification protein rimK-like family member A; Ad-RIMKLA. Adenoviral RIMKLA; GFP. Green fluorescent protein; PKM2. Pyruvate kinase M2; PTP1B. Protein-tyrosine phosphatase 1B; rRIMKLA. recombinant RIMKLA; DAPI. 4,6’-diamidino-2-phenylindole; P. PTP1B-IN-2; C. C3K; ATP. Adenosine triphosphate.

Phosphorylation of PTP1B at Tyr66 has been shown to mediate PKM2 dephosphorylation at Tyr105 [7], a key determinant of PKM2 activation [8]. In VSMCs, *RIMKLA* overexpression led to a reduction in PKM2 phosphorylation at Tyr105 (Fig. 5**a**). However, incubation with recombinant RIMKLA (rRIMKLA) did not affect PKM2 phosphorylation *in vitro* (Fig. 5**b**), suggesting that RIMKLA does not dephosphorylate PKM2 directly. Instead, the RIMKLA-induced dephosphorylation of PKM2 was reversed by a PTP1B inhibitor (Fig. 5**c**), indicating that RIMKLA exerts its effect through PTP1B. *RIMKLA* overexpression increased PTP1B phosphorylation at Tyr66, whereas silencing *RIMKLA* reduced it, with corresponding changes observed in PKM2 Tyr105 phosphorylation (Fig. 5**d, e**). Notably, incubation with rRIMKLA led to direct phosphorylation of PTP1B at Tyr66 *in vitro* (Fig. 5**f**), indicating that RIMKLA functions as an upstream kinase of PTP1B.

Co-IP was conducted to confirm the interaction between RIMKLA and PTP1B (Fig. 5**g**). ZDOCK docking analysis revealed that RIMKLA possesses a classical ATP-grasp domain, characteristic of protein kinases, and that the Tyr66 site of PTP1B is positioned near this domain at the RIMKLA-PTP1B interaction interface (Fig. 5**h**). These findings suggest that RIMKLA not only functions as an upstream kinase for PTP1B but it may also act as a scaffold facilitating the interaction between PTP1B and PKM2. Co-IP analysis confirmed the interaction between PTP1B and PKM2, with *RIMKLA* overexpression enhancing this interaction and *RIMKLA* silencing reducing it, without affecting their expression in VSMCs (Fig. 5**i, j**).

PTP1B-PKM2 colocalization was also upregulated in the mesenteric arterial media (Fig. 5**k**) and thoracic aorta of salt-sensitive hypertensive Dahl/SS rats (**Additional file 2:** Fig. S12c). Similarly, in *RIMKLA*-overexpressing mice (**Additional file 2:** Fig. S12d) and cultured VSMCs (**Additional file 2:** Fig. S12e), PTP1B-PKM2 colocalization was increased. Interestingly, PTP1B expression remained unchanged in the mesenteric arteries of hypertensive rats compared with control rats (**Additional file 2:** Fig. S12f). Functionally, the inhibition of either PTP1B or PKM2 suppressed the RIMKLA-induced increases in pyruvate kinase activity (Fig. 5**l**, left) and pyruvate production (Fig. 5**l**, right) in VSMCs.

**Fig. 6:**
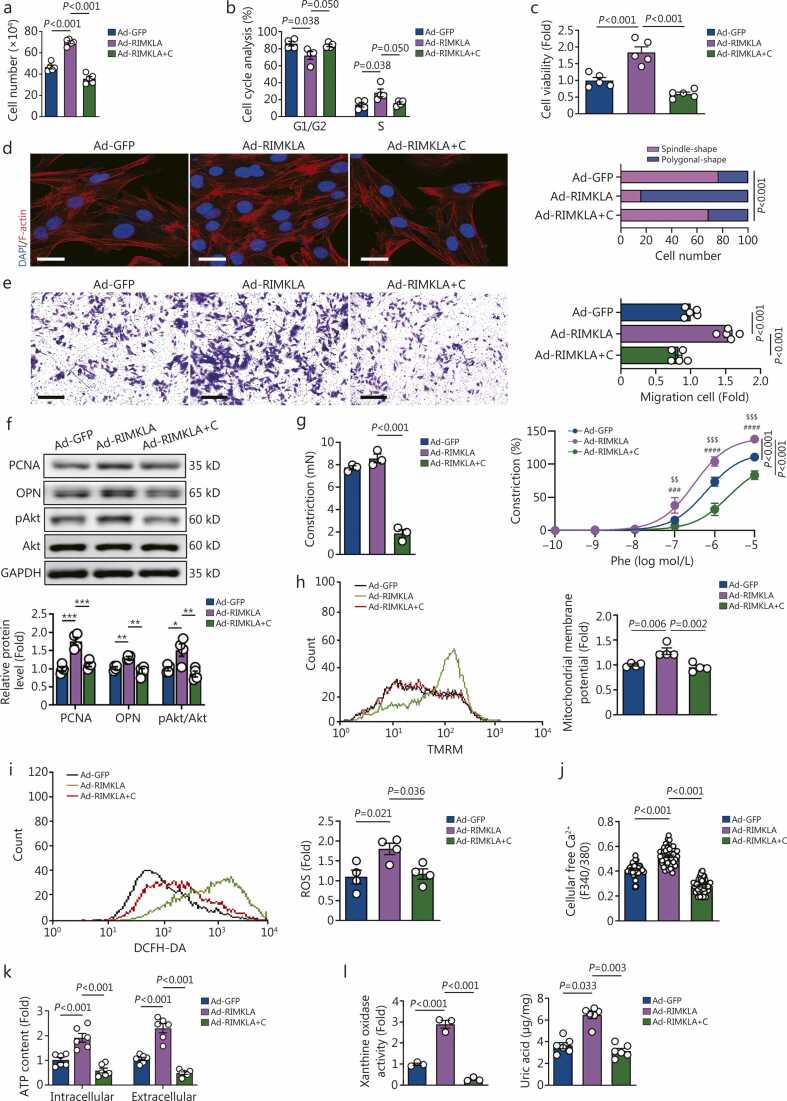
Inhibition of PKM2 blocks RIMKLA-induced VSMC phenotype switch. Treatment with PKM2 inhibitor (C3K, 0.5 μmol/L) restrained RIMKLA-induced proliferation of VSMCs as evaluated by assays of cell number counting (a), cell cycle (b), cell viability (c), and cell morphology (d) (n=4–5). For (d), scale bar=25 μm, data were obtained from 100 cells from three independent experiments. e Treatment with PKM2 inhibitor (C3K, 0.5 μmol/L) reversed RIMKLA-induced migration of VSMCs as assessed by cell transwell assay (n=5). Scale bar=200 μm. Inhibition of PKM2 suppressed RIMKLA-induced increase in PCNA and OPN protein levels, and Akt phosphorylation in VSMCs (f, n=4), as well as reduced RIMKLA-induced arterial vasoconstriction of SD rats (g, n=3). ^⁎^P<0.05, ^⁎⁎^P<0.01, ^⁎⁎⁎^P<0.001 (f). ^$^P<0.05^, $$^P<0.01, ^$$$^P<0.001, Ad^-^RIMKLA vs. Ad-GFP; ^##^P<0.01, ^###^P<0.001, ^####^P<0.0001, Ad-RIMKLA+C vs. Ad-RIMKLA (g^).^ Treatment with PKM2 inhibitor (C3K, 0.5 μmol/L) reversed RIMKLA-promoted increase in mitochondrial membrane potential (h) and ROS production (i) in VSMCs (n=4). j Inhibition of PKM2 reversed RIMKLA-induced increase in cellular Ca^2+^ levels in VSMCs. Data were obtained from more than 100 cells from 5 independent experiments. k Treatment with PKM2 inhibitor reversed the increased intracellular and extracellular ATP content induced by Ad-RIMKLA treatment in VSMCs (n=6). l Treatment of PKM2 inhibitor suppressed the increase in xanthine oxidase activity and uric acid production induced by overexpression of RIMKLA in VSMCs (n=3–6). Chi‑square test for (d); two‑way ANOVA with Bonferroni and Tukey’s corrections for (g-right); Kruskal‑Wallis test with Dunn’s post hoc for (j, l-right) and one‑way ANOVA with Bonferroni post hoc for others. VSMC. Vascular smooth muscle cell; RIMKLA. Ribosomal modification protein rimK-like family member A; Ad-RIMKLA. Adenoviral RIMKLA; GFP. Green fluorescent protein; PKM2. Pyruvate kinase M2; P. PTP1B-IN-2; C. C3K.

To further elucidate the role of PKM2 in RIMKLA-mediated VSMC phenotype switch, the PKM2 inhibitor compound 3k (C3K) was used. Treatment with C3K effectively inhibited RIMKLA-induced VSMC proliferation, as demonstrated by reductions in cell number (Fig. 6**a**), altered cell cycle progression (Fig. 6**b**), decreased cell viability (Fig. 6**c**), changes in cell morphology (Fig. 6**d**), and suppressed cell migration (Fig. 6**e**). Additionally, C3K treatment reduced the expression of PCNA and OPN and inhibited Akt activation (Fig. 6**f**). Functionally, C3K reversed RIMKLA-induced vasoconstriction in rat arteries (Fig. 6**g**). Furthermore, treatment with the PKM2 inhibitor blocked the RIMKLA-induced increases in mitochondrial membrane potential (Fig. 6**h**), ROS production (Fig. 6**i**), intracellular Ca^2+^ concentration (Fig. 6**j**), ATP production and secretion (Fig. 6**k**), xanthine oxidase activity (Fig. 6**l**, left), and uric acid production (Fig. 6**l**, right) in VSMCs. Overall, these results suggest that RIMKLA acts as a kinase-like scaffold, activating PKM2 and regulating VSMC phenotype switch.

### 3.7 VSMC-specific deletion of PKM2 abolishes RIMKLA-induced hypertension

To investigate the role of PKM2 in RIMKLA-mediated hypertension *in vivo*, VSMC-specific *PKM2* knockout mice were generated using the Cre-Loxp system (**Additional file 2:** Fig. S13a). Successful *PKM2* deletion in VSMCs was confirmed by the expression of *Loxp* and *Cre* (**Additional file 2:** Fig. S13b), as well as the reduction in *PKM2* mRNA (**Additional file 2:** Fig. S13c) and protein expression in the mesenteric artery (**Additional file 2:** Fig. S13d). Under physiological conditions, no significant differences were observed in cardiac systolic function (**Additional file 2:** Fig. S13e**-g**), maximal abdominal aortic diameter (**Additional file 2:** Fig. S13h), cardiac morphology, heart weight, or heart to body weight ratio (**Additional file 2:** Fig. S13i) between PKM2^VSMC−/−^ mice and PKM2^flox/flox^ controls.

**Fig. 7:**
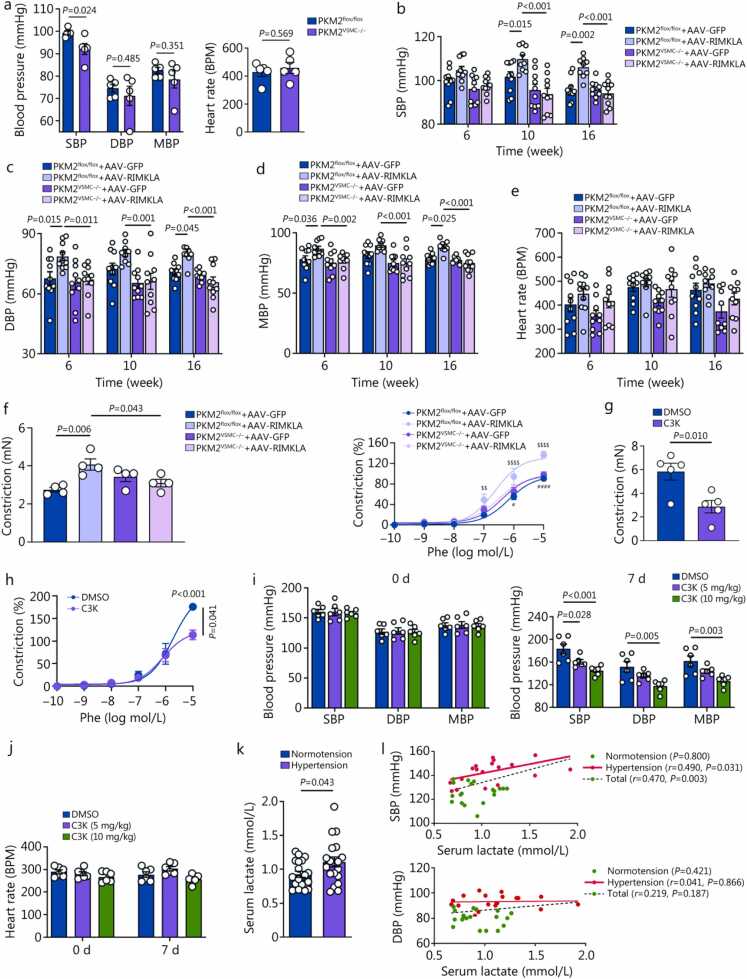
Treatment of PKM2 inhibitor reduces blood pressure of SHR. a VSMC-specific deletion of PKM2 reduced SBP in 8-week-old male mice with no significant difference in DBP and MBP as well as heart rates when compared to control mice (n=5). VSMC-specific overexpression of RIMKLA failed to increase blood pressure levels in PKM2^VSMC−/−^ mice (b-d), and had no effect on heart rates in either PKM2^VSMC−/−^or PKM2^flox/flox^ mice (e) (n=10). f AAV-RIMKLA injection increased the contractions of thoracic arteries of PKM2^flox/flox^ mice but not PKM2^VSMC−/−^ mice (n=4). ^$$^P<0.01, ^$$$$^P<0.0001, PKM2^flox/flox^+AAV-RIMKLA vs. PKM2^flox/flox^+AAV-GFP; ^#^P<0.05, ^####^P<0.0001, PKM2^VSMC−/−^+AAV-RIMKLA vs. PKM2^flox/flox^+AAV-RIMKLA. Preincubation with PKM2 inhibitor reduced the vasoconstriction of SHR arteries to potassium (g) and Phe (h) (n=5). Superior mesenteric arteries were incubated with PKM2 inhibitor (C3K, 30 μmol/L) for 6 h before vasoconstriction and vasodilatation measurement. i Treatment with PKM2 inhibitor dose-dependently reduced blood pressure of SHR (n=6). SHR rats were treated with PKM2 inhibitor C3K at the daily dose of 5 mg/kg or 10 mg/kg body weight (in DMSO) for 7 d via intraperitoneal injection, respectively, and control SHR rats were treated with DMSO. Blood pressure and heart rate of rats were measured by the tail-cuff method before and after treatment. j Treatment with PKM2 inhibitor on the heart rates of SHR rats (n=6). k Serum lactate was increased in hypertensive patients compared to normotensive controls (n=19). l Serum lactate was positively correlated with SBP (r=0.490, 95% CI 0.052–0.770), with no correlation with DBP in hypertensive patients. In normotensive controls, serum lactate did not correlate with blood pressure (n=19). P‑values were analyzed by unpaired t‑test (a, g, k), one‑way ANOVA with Bonferroni post hoc (i, j), and Pearson correlation (l). Others are determined by two‑way ANOVA with Bonferroni and Tukey’s corrections. SBP. Systolic blood pressure; DBP. Diastolic blood pressure; MBP. Mean blood pressure; RIMKLA. Ribosomal modification protein rimK-like family member A; Ad-RIMKLA. Adenoviral RIMKLA; GFP. Green fluorescent protein; PKM2. Pyruvate kinase M2; Phe. Phenylephrine; VSMCs. Vascular smooth muscular cells.

At baseline, PKM2^VSMC−/−^ mice (8 weeks old) exhibited a slight reduction in SBP of approximately 10 mmHg compared with PKM2^flox/flox^ mice, with no significant differences in DBP, MBP, or heart rate (Fig. 7**a**). Following transduction with AAV-RIMKLA for 1 month in PKM2^VSMC−/−^ and PKM2^flox/flox^ mice to overexpress *RIMKLA* in VSMCs, blood pressure was measured (**Additional file 2:** Fig. S14a). Successful VSMC-specific *PKM2* deletion and *RIMKLA* overexpression were confirmed by reduced PKM2 protein expression and increased RIMKLA protein expression in the renal and mesenteric arteries (**Additional file 2:** Fig. S14b**, c**). *RIMKLA* overexpression significantly increased blood pressure in control PKM2^flox/flox^ mice (Fig. 7**b-d**). In contrast, VSMC-specific *PKM2* deletion effectively abolished the RIMKLA-induced increases in SBP (by approximately 20 mmHg at 10 and 16 weeks post-injection), as well as DBP and MBP (each by approximately 15 mmHg at 6, 10 and 16 weeks post-injection), without affecting heart rate (Fig. 7**b-e**).

*PKM2* deletion in VSMCs blocked RIMKLA-induced vasoconstriction in response to potassium (Fig. 7**f**, left) and phenylephrine (Fig. 7**f**, right), without affecting vasodilation (**Additional file 2:** Fig. S14d). Notably, there were no significant differences in arterial remodeling (**Additional file 2:** Fig. S15a) or cardiac phenotypes, heart weight and heart to body weight ratio (**Additional file 2:** Fig. S15b**, c**) between PKM2^VSMC−/−^ and PKM2^flox/flox^ mice, regardless of *RIMKLA* overexpression. These findings provide strong evidence that PKM2 is a critical downstream target of RIMKLA, and its deletion prevents RIMKLA-induced vasoconstriction and hypertension, underscoring the functional importance of PKM2 in RIMKLA-mediated vascular pathophysiology.

### 3.8 PKM2 inhibitor attenuates blood pressure of SHR

The results of this study suggest that PKM2 linked RIMKLA to VSMC dysfunction/hypertension. Preincubation with C3K significantly and dose-dependently reduced vasoconstriction induced by potassium and phenylephrine **(**Fig. 7**g, h; Additional file 2:** Fig. S16a) in the mesenteric arteries of both SD and SHR rats, without affecting vasodilation (**Additional file 2:** Fig. S16b**, c**).

*In vivo*, intraperitoneal injection of C3K [5 mg/(kg·d) and 10 mg/(kg·d)] for 1 week significantly reduced SBP, DBP, and MBP in SHR rats (Fig. 7**i**), with no significant change in heart rate in either C3K‑treated group relative to the DMSO control **(**Fig. 7**j)**. C3K at 10 mg/(kg·d) also attenuated renal and thoracic artery remodeling, as evidenced by decreased renal artery medial layer thickness (**Additional file 2:** Fig. S17a), and reduced the ratio of thoracic artery medial thickness to lumen diameter (**Additional file 2:** Fig. S17b), along with a declining trend in mesenteric artery medial thickness (**Additional file 2:** Fig. S17c). Additionally, the PKM2 inhibitor C3K exerted a dose-dependent effect on VSMC phenotype switch by promoting the contractile phenotype, as shown by the increase in CNN1 protein expression (**Additional file 2:** Fig. S18, top) and restraining the synthetic phenotype, as indicated by the decrease in OPN expression in renal artery VSMCs (**Additional file 2:** Fig. S18, bottom). Collectively, the PKM2 inhibitor could potentially be used to treat human hypertension and vascular diseases.

In addition to its role in pyruvate metabolism within the TCA cycle, pyruvate can be converted to lactate. Several lines of evidence have demonstrated the positive correlations between serum lactate concentration and metabolic disease [21]. Particularly, SBP and DBP were reported to be positively associated with plasma lactate concentration in an observational study (*r*=0.36, *P*=0.01) [22]. Consistent with this, *RIMKLA* overexpression in VSMCs was found to increase lactate content in the present study (**Additional file 2:** Fig. S19a). In hypertensive rats, serum lactate, but not pyruvate was elevated compared with control rats (**Additional file 2:** Fig. S19b). Similarly, VSMC-specific overexpression or deletion of *RIMKLA* in mice resulted in significant changes in serum lactate concentration, with overexpression increasing and deletion decreasing the lactate concentration, while the serum pyruvate concentration remained largely unaffected (**Additional file 2:** Fig. S19c**, d**). Importantly, the serum lactate concentration was increased in patients with hypertension compared with normotensive controls (Fig. 7**k**) (the basal clinical characteristics are provided in **Additional file 1:** Table S7) and was positively correlated with SBP but not DBP in patients with hypertension (Fig. 7**l**). Overall, these findings suggest that activated RIMKLA expression in VSMCs increased serum lactate level, which could be a potential risk factor for hypertension in humans.

## 4 Discussion

**Fig. 8:**
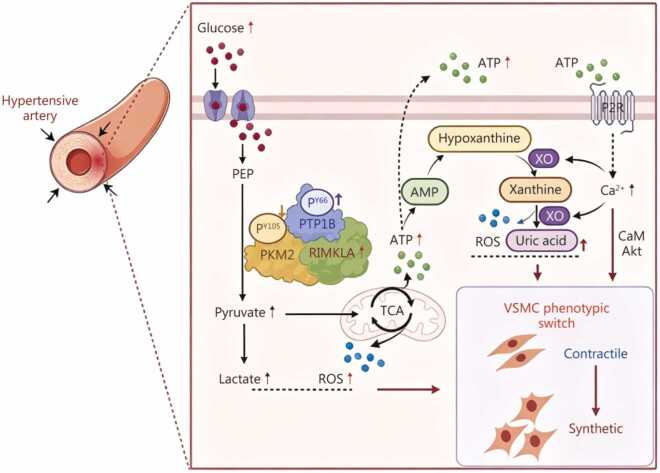
Proposed model for RIMKLA in the pathogenesis of hypertension. In VSMCs, RIMKLA phosphorates and recruits PTP1B to activate PKM2, thereby promoting VSMC glucose metabolism to trigger ATP synthesis and ROS production. The released ATP stimulates VSMC phenotype switch from contractile phenotype to proliferative phenotype by activating P2 receptor (P2R) to elevate cellular Ca^2+^ levels, ultimately regulating vasoconstriction and blood pressure. Additionally, the increased Ca^2+^ activates xanthine oxidase (XO), thus promoting the conversion of xanthine to uric acid, with large amount of ROS production. Activation of P2R signaling, along with increased uric acid and ROS production, collectively mediates the pathogenetic effects of RIMKLA on VSMC phenotype switch and hypertension. Created in BioRender with the publication license (agreement number: KA29AJRD98). PEP. Phosphoenolpyruvate; ROS. Reactive oxygen species; TCA. Tricarboxylic acid cycle; VSMCs. Vascular smooth muscular cells; PKM2. Pyruvate kinase M2; PTP1B. Protein-tyrosine phosphatase 1B; ATP. Adenosine triphosphate; AMP. Adenosine monophosphate; CaM. Calmodulin; Akt. Protein kinase B.

The precise regulation of PKM2 activity, a key glycolytic enzyme, is critical for maintaining normal glucose metabolism. In this study, we have uncovered a novel mechanism regulating PKM2 activity in VSMCs and subsequently elucidated its implications in the pathogenesis of hypertension. *RIMKLA* gene, identified according to a high GIC score, played a pivotal role in this mechanism. In the present study, RIMKLA orchestrates a cascade wherein it directly phosphorylates PTP1B, recruiting it to dephosphorylate and activate PKM2. This intricate process promotes glucose metabolism, resulting in ATP synthesis and ROS production in VSMCs. The consequential release of ATP and heightened ROS levels induce a phenotypic transition in VSMCs from contractile to synthetic, intricately influencing arterial constriction and blood pressure regulation (Fig. 8).

The dysregulation of glucose metabolism is closely associated with hypertensive conditions and vascular diseases. Recent studies have suggested that the reprogramming of glucose metabolism, shifting from mitochondrial OXPHOS to glycolysis, may contribute to VSMC phenotype switch [6], [23] and vascular diseases [24], [25]. For instance, in primary pulmonary artery endothelial cells and pulmonary artery smooth muscle cells derived from patients with pulmonary arterial hypertension and animal models, altered glucose metabolism was observed, leading to reduced oxidative glucose metabolism [26]. In addition, metabolic shifting from OXPHOS towards glycolysis, and ultimately to lactate, has been implicated in macrophage polarization and cardiac remodeling [27]. Dysregulated macrophage polarization due to abnormal glycolysis exacerbates hypertension-induced cardiac injury and perturbs calcium homeostasis. Ji *et al*. [28] reported that disrupted mitochondrial homeostasis results in enhanced mitochondrial membrane potential and ROS production, contributing to VSMC phenotype switch. Consistent with these findings, our study reveals that *RIMKLA* overexpression in VSMCs remarkably increased mitochondrial membrane potential, ATP synthesis, ROS production, and cellular calcium concentration, thereby promoting VSMCs phenotype switch.

PKM2, a pyruvate kinase subtype localized in proliferative cells such as embryonic cells, stem cells, proliferative VSMCs, and tumor cells [29]. Previous studies have highlighted the multifaceted role of PKM2 in vascular remodeling [4], [5] and its association with various cellular processes, including energy supply to tumor cells, epithelial-mesenchymal transition, cell invasion, metastasis, and proliferation [30], [31]. Notably, a recent finding indicated the involvement of PKM2 in neovascularization through the regulation of glycolysis, mitochondrial dynamics, and fusion in vascular resident endothelial progenitor cells (VR-EPCs) [32]. Enhanced VR-EPC proliferation and PKM2 activity were observed with PKM2 agonists, while these processes were blocked by PKM2 inhibitors. Complementing these observations, other research has revealed that PKM2 plays a role in mouse VSMC phenotype switch and neointimal hyperplasia development [5]. Consistent with these studies, our investigation aligns with the observation that deletion of *PKM2* in VSMCs reduced injury-induced neointimal hyperplasia by inhibiting VSMC proliferation and migration. Our study further extends these insights by demonstrating that a PKM2 inhibitor suppressed RIMKLA-induced VSMC phenotype switch. Additionally, our examination of hypertensive animals revealed elevated PKM2 activity in the thoracic aorta, and the administration of a PKM2 inhibitor resulted in reduced vasoconstriction and blood pressure in SHR rats. Collectively, these findings position PKM2 as a pivotal mediator of RIMKLA’s effects on VSMC glucose metabolism, vascular tone regulation, and blood pressure modulation.

Central to our study is the intricate regulation of PKM2 enzymatic activity, a process influenced by alternative PKM2 expression [33], [34], splicing [6], and various post-translational modifications (PTMs) including phosphorylation and acetylation [35]. PKM2 dimer/tetramer formation may also play a role in PKM2 activity [36]. The dimeric form of PKM2 exhibits lower glycolytic activity but can translocate to the nucleus, functioning as a nuclear coactivator regulating gene expression. When PKM2 exists in the tetrameric state, it exhibits high affinity for its substrate, phosphoenolpyruvate (PEP), thereby possessing elevated pyruvate kinase activity. The Tyr residue Y105 of PKM2 is phosphorylated in various human tumors [37]. Generally, PKM2 Tyr phosphorylation is modulated by Tyr kinases and phosphatases. Notably, PTP1B, a widely expressed non-receptor Tyr-specific phosphatase, has emerged as a pivotal modulator of PKM2 activity by phosphorylation at Tyr105 of PKM2 [7], [38]. PTP1B itself undergoes a spectrum of reversible and irreversible PTMs, with phosphorylation at specific sites exerting a pronounced impact on its phosphatase activity. The intricate interplay of these modifications is exemplified by Akt phosphorylation at Ser50, which hampers PTP1B activity [39], whereas insulin receptor Tyr kinase-induced phosphorylation at Tyr66, Tyr152, and Tyr153 activates PTP1B [40]. Particularly noteworthy is the significant role of Tyr66 phosphorylation in insulin responsiveness, leading to heightened PTP1B activity and subsequent negative regulation of the insulin pathway [40]. The present study highlighted the pivotal role of RIMKLA in orchestrating the direct phosphorylation of PTP1B at Tyr66 site, culminating in the subsequent dephosphorylation and activation of PKM2. Consequently, our findings address a critical void in the current understanding by delineating the role of PTP1B-mediated Tyr phosphorylation in modulating PKM2 activity within VSMCs, contributing important insights to our comprehension of cardiovascular disease mechanisms.

A salient revelation of this study pertains to the involvement of uric acid and ROS in mediating the phenotypic transformation of VSMCs induced by RIMKLA. Uric acid, a final product of ATP catabolism, undergoes renal metabolism for excretion. Impairment in uric acid metabolism culminates in hyperuricemia, characterized by elevated circulating uric acid concentrations, which is closely associated with cardiovascular disease, particularly hypertension [41]. ATP catabolism into uric acid generates substantial ROS, and the low blood solubility of uric acid in blood facilitates crystal deposition on vessel walls. Monosodium urate crystals interact with ROS, causing severe vascular endothelial damage and activating inflammatory factors, initiating an inflammatory cascade response [42].

Previous studies have demonstrated that elevated plasma uric acid promotes VSMC proliferation through mitogen-activated protein kinase (MAPK) activation and upregulates monocyte chemoattractant protein-1 expression by activating MAPK and cyclooxygenase-2 [43], [44]. This cascade leads to monocyte accumulation, adherence to VSMCs, and inflammatory cytokines secretion, ultimately causing significant vascular damage. Our study indicates that *RIMKLA* overexpression increases xanthine oxidase activity and uric acid content. Inhibiting xanthine oxidase or scavenging ROS inhibits RIMKLA-induced VSMC proliferation. Thus, our hypothesis posits that RIMKLA-induced VSMC proliferation, migration, and phenotype switch may be triggered not only by enhanced VSMC glucose metabolism and ATP synthesis through PKM2 activation but also by increased uric acid and ROS production.

Heightened uric acid and ROS may induce VSMC proliferation by activating signaling pathways, such as MAPK signaling. This activation, coupled with increased mitochondrial membrane potential due to glycolysis, leads to additional ROS production through xanthine oxidation [45]. RIMKLA-induced phosphorylation of PTP1B and its recruitment for PKM2 activation amplify glycolysis, elevating mitochondrial membrane potential, ATP synthesis, and ROS production. In turn, the released ATP acts on P2 receptors to raise cellular calcium concentration, thereby activating calmodulin, Akt, and xanthine oxidase activity. Released ATP is likely to activate a range of purinergic P2 receptors, such as P2X7 [46], P2Y1 [15], and P2Y2 [47], thereby modulating intracellular calcium concentration and VSMC phenotype switch. The ensuing increase in xanthine oxidase activity stimulates uric acid production, accompanied by additional ROS generation beyond the mitochondria. This intricate molecular cascade provides novel insights into the mechanisms underlying the excessive production and accumulation of uric acid in conditions such as hyperhomocysteinemia and Ang II elevation. The established link between increased mitochondrial membrane potential, ROS, and RIMKLA activation signifies a novel pathway contributing to ROS production and, consequently, vascular dysfunction.

## 5 Limitations

To minimize the confounding effects of estrogen, our study primarily used male animals. Blood pressure in females fluctuates across the menstrual cycle, which complicates experimental monitoring. Moreover, female ovarian hormones, particularly estrogen, typically confer vasoprotection by inhibiting VSMC phenotype switch. Consequently, the pathological effects of RIMKLA may be attenuated in females due to this hormonal influence. The use of only male animals in this study may limit the generalizability of our findings. Future work is essential to validate the results in female animal models.

The exact molecular mechanism by which RIMKLA recruited PTP1B to activate downstream signaling remains unknown, which requires systematic evaluation. In the current study, we observed that in VSMCs, RIMKLA interacted with PTP1B and subsequently activated PKM2 to exert biological functions in the pathogenesis of hypertension. It is necessary to check whether this also applies in other cardiovascular cell types, such as endothelial cells and cardiomyocytes, as well as in the progression of other cardiovascular diseases.

PKM2 is ubiquitously expressed in proliferating cells and plays a pivotal role in regulating cellular metabolism. Therapeutic targeting of the RIMKLA-PKM2 axis in VSMCs shows promise for treating hypertension; however, potential off‑target effects in other tissues warrant systematic evaluation. Although a comprehensive assessment of long-term efficacy, durability, and safety is required before clinical translation, we conducted a proof-of-concept study to test the translational potential of targeting the RIMKLA-PKM2 axis using an acute/sub-acute (1-week) pharmacological intervention with the PKM2 inhibitor C3K and demonstrated effective reversal of established vascular phenotypes. This finding provides foundational support for future development, pending further chronic administration studies. The crystal structure of the RIMKLA-PTP1B-PKM2 complex should be established and evaluated, which will provide a solid foundation for further targeted drug discovery.

We also note that the data for *in vitro* kinase activity were obtained using commercial recombinant RIMKLA protein without a parallel kinase‑dead mutant control. Therefore, definitive structural and catalytic validation of the RIMKLA kinase domain, including site‑directed mutagenesis of its predicted ATP‑binding residues, remains an important direction for future investigation.

## 6 Conclusions

Our study highlights RIMKLA as a previously unrecognized protein kinase orchestrating PTP1B phosphorylation and its subsequent recruitment to activate PKM2. This molecular cascade acts as a regulatory mechanism influencing glucose metabolism, thereby impacting VSMC function and blood pressure regulation. The identified overactivation of the RIMKLA-PKM2 pathway in VSMCs serves as a novel pathogenic mechanism underlying hypertension and vascular diseases. Particularly, PKM2 assumes a central role linking RIMKLA to VSMC phenotype switch, as well as vascular tension regulation. Our findings are relevant to the potential therapeutic application of PKM2 inhibitors for human hypertension and vascular diseases.

## Abbreviations

Akt: Protein kinase B
Ang II: Angiotensin II
ATP: Adenosine triphosphate
Ach: Acetylcholine
BHMT1: Betaine-homocysteine S-methyltransferase 1
CNN1: Calponin 1
C3K: Compound 3k (PKM2 inhibitor)
ECAR: Extracellular acidification rate
FB: Febuxostat (xanthine oxidase inhibitor)
GIC: Gene Importance Calculator
GWAS: Genome-wide association studies
GST: Glutathione S-transferase
Hcy: Homocysteine
MAPK: Mitogen-activated protein kinase
MS: Mass spectrometry
MOI: Multiplicity of infection
NAC: N-acetyl-L-cysteine
OPN: Osteopontin
OCR: Oxygen consumption rate
OXPHOS: Oxidative phosphorylation
Oligo: Oligomycin
PBS: Phosphate buffer saline
Phe: Phenylephrine
PCNA: Proliferating cell nuclear antigen
PKM2: Pyruvate kinase M2
PEP: Phosphoenolpyruvate
PTP1B: Protein-tyrosine phosphatase 1B
PTMs: Post-translational modifications
RIMKLA: Ribosomal modification protein rimK-like family member A
ROS: Reactive oxygen species
SNP: Sodium nitroprusside
SD: Sprague-Dawley
SHR: Spontaneous hypertension rat
TCA: Tricarboxylic acid cycle
VSMC: Vascular smooth muscle cell

## Ethics approval and consent to participate

Human arteries have been obtained in our previous study [15], and the study was approved by the Ethics Review Board of Fuwai Hospital, Beijing, China (2023-2159). The serum samples of normotension or hypertension were provided by Beijing Chao-Yang Hospital, Capital Medical University. The demographic characteristics were shown in **Additional file 1:** Table S7. The application for patient information was approved by the Research Ethics Committee Beijing Chao-Yang Hospital (2024-12-11-5). Informed consents were obtained from each participant. All animal care and experimental protocols complied with the Animal Management Rules of the Ministry of Health of the People’s Republic of China and the guide for the Care and Use of the Laboratory Animals of the Peking University. Animal experiments were proven by the Institutional Animal Care and Use Committee of Peking University Health Science Center (BCJB0037).

## CRediT authorship contributions statement

RX and WJL researched data and contributed to the discussion. XRZ, HY, XL, SH, CQH, YTH, RFM, and YJC provided the technical assistance and animal preparation. JL provided human serum samples. JCY and RX wrote the manuscript. JCY, RX, BG, and ZZC designed the study, and revised/edited manuscript. BG, ZZC, and QHC contributed to the discussion. QHC and his group assisted solving the statistical issues. All authors read and approved the manuscript.

## Funding

This work was supported by the Noncommunicable Chronic Diseases-National Science and Technology Major Project (2024ZD0523100), the National Natural Science Foundation of China (82300501, 82230024, 82025008, 82370448, 82470461, 82070844, 82300957, 82400960), and the Peking University Medicine Seed Fund for Interdisciplinary Research (BMU2023YFJHMX002, supported by the Fundamental Research Funds for the Central Universities).

## Competing interests

The authors declare that they have no conflict of interests.

## Data Availability

All data and raw gel images are included with the paper. All materials are available from the lead contact upon reasonable request. Gene Importance Calculator (GIC) (http://www.cuilab.cn/gic) predicts *RIMKLA* gene with high essentiality (**Additional file 1:** Table S1). Raw data for LC-MS analysis of untargeted metabolomics (**Additional file 1:** Tables S4, S5) and potential RIMKLA interacting molecules (**Additional file 1:** Table S6) are included in Supplementary Information. There is no restriction on data availability. The AlphaFold prediction of RIMKLA (AF-Q8IXN7-F1) was used as initial models for protein docking, and the PDB formats of PKM2 (6JFB) and PTP1B (1A5Y) structural domains were downloaded from the Protein Data Bank PDB database (http://www.rcsb.org/). Other publicly available datasets were used for analyzing conserved *RIMKLA* gene sequence (http://genome.ucsc.edu/) and GWAS catalog for *RIMKLA* gene (https://www.ebi.ac.uk/gwas/).

## Footnotes

Appendix A Supplementary data associated with this article can be found in the online version at doi:10.1016/j.mmr.2026.100056.
