# Supplementary material for: RIMKLA promotes hypertension by activating PKM2 to trigger VSMC phenotype switch
Source: Mil Med Res. 2026 Jul 24;13(1):100056. doi: 10.1016/j.mmr.2026.100056 (PMC13420487; doi:10.1016/j.mmr.2026.100056)
Supplement: Supplementary file 2 — Supplementary material [file mmc2.pdf]

Methods

Primary culture of vascular smooth muscle cells

Aortic smooth muscle cells were isolated from rats and mice and cultured in DMEM medium supplemented with 20% fetal bovine serum (FBS), 2 mmol/L L-glutamine, 100 U/ml penicillin, and 10 mg/ml streptomycin as detailed previously [1]. All experimental procedures were conducted in an incubator at a temperature of 37 ℃, in an atmosphere of 95% air and 5% CO_2. The vascular smooth muscle cells (VSMCs) with passage numbers 3 to 8 were used in the study. Cells were infected with 50 multiplicity of infection (MOI) Ad-green fluorescent protein (GFP) or Ad-ribosomal modification protein rimK-like family member A (RIMKLA) for 24 h in the absence or presence of pyruvate kinase M2 (PKM2) inhibitor (C3K, 0.5 μmol/L; #S8616, Selleck, USA), adenosine triphosphate (ATP)-P2 receptor inhibitor (suramin, 40 μmol/L; #ab120422, Abcam, UK), protein-tyrosine phosphatase 1B (PTP1B) inhibitor (PTP1B-IN-2, 15 μmol/L; #S0119, Selleck, USA), reactive oxygen species (ROS) scavenger (NAC, 10 μmol/L; #ST1546, Beyotime, China) or xanthine oxidase inhibitor (FB, 10 μmol/L; #SF1114, Beyotime, China) before experimental assays. Cells were transfected with 50 nmol/L siRIMKLA (Invitrogen, USA) via a transfection reagent (#KX0110049, Biodragon, Beijing, China) for 24 h before experimental assays.

Immunofluorescence staining

The procedure was performed as previously described [1]. In brief, the arteries of humans or animals were harvested and fixed overnight in 4% PFA at 4 °C, then transferred to 30% glucose solution and incubated at 4 ℃ until tissues sink. Nonspecific binding sites were blocked in 10% goat serum blocking solution for 1 h. And then incubated with the following antibodies: mouse anti-α-smooth muscle actin antibody (1:200, #A5228, Sigma, USA), rabbit anti RIMKLA (1:100, #PA5-101852, Thermo Fisher Scientific, USA), rabbit anti PKM2 (1:100, #4053, CST, USA) or rabbit anti PTP1B (1:100, #ab244207, Abcam, UK) at 4 ℃ overnight. After washing with phosphate buffer saline (PBS), the tissues were incubated with the secondary antibody: Alexa Fluor 594®-conjugated goat anti-mouse IgG (#ZF-0513, ZSGB-Bio, China) and Alexa Fluor 647®-conjugated goat anti-rat IgG (#35104, Yeasen, China) at 37 ℃ for 2 h, and then stained with 4,6'-diamidino-2-phenylindole (DAPI; #D1306, Invitrogen, USA) to label cell nuclei. The fluorescence images were obtained using confocal microscopy (TCS-SP8 STED, Leica, Germany).

Cellular immunofluorescence

Cells seeded on coverslips were treated with Ad-RIMKLA with or without the above-mentioned inhibitors for 24 h (Ad-GFP served as control treatment). The coverslips were incubated with the relevant antibodies

at 4 ℃ overnight. After being washed with PBS, the cells were incubated with the secondary antibody at 37 °C for 1 h, and then stained with DAPI to label cell nuclei. Then, the coverslips were mounted on glass slides using 50% glycerol in PBS. Images were visualized by fluorescence microscopy using a confocal laser scanning microscope (TCS-SP8 STED, Leica, Germany).

Cell viability assay

Cell viability was measured by methylthiazolyldiphenyl-tetrazolium bromide (MTT) assay as detailed previously [1]. Briefly, MTT (#ST316, Beyotime, China) was added into the culture medium to the final concentration of 0.5 mg/ml, and then the cells were incubated for 4 at 37 ℃ in an incubator. The absorbance at 490 nm was measured using an automatic plate reader (Varioskan™ LUX, Thermo, USA). The average absorbance reflected cell viability with the data normalized to the control group.

Cell proliferation and cell cycle analysis

The procedure was performed as previously described [1]. In general, VSMCs were harvested and washed twice with FACS buffer and treated with 200 μg/ml RNase A (#R4642, Sigma, USA) for 1 h (37 ℃), then stained for DNA labeling with 40 μg/ml propidium iodide solution for 20 min at 4 ℃. The stained cells were transferred to flow cytometry tubes for cell cycle analysis in a flow cytometer (Becton Dickinson, USA).

Cell migration and transwell assay

After treatment for 24 h, VSMCs were harvested and resuspended in serum-free DMEM. Ensuring similar cell numbers in different treatments via Neubauer counting chamber. Then the counted cells (100 μl) were transferred to the upper chamber of the Transwell plate (#CLS3422, Corning, USA), with 600 μl DMEM containing 20% FBS in the lower chamber for 12–24 h. After washing twice with PBS, the transwell chambers were fixed with 4% paraformaldehyde (PFA) for 30 min and stained with 0.1% crystal violet solution for 20 min. Images were visualized by microscopy. 33% acetic acid solution could be used to elute cells on the surface of the Transwell chamber. The absorbance at 570 nm was measured using an automatic plate reader (Varioskan™ LUX, Thermo, USA). The average absorbance reflected cell migration, with the data normalized to the control group.

Cytoplasmic calcium assay

The procedure was performed as previously described [1]. In brief, VSMCs were seeded in 6-well plates (each well was pre-placed with a 24 mm×24 mm coverslip). After treatment for 24 h, 1 μmol/L Fura-2 AM (as molecular probe) (#F1221, Invitrogen, USA) was added to each well for 30 min. Then washed

wells three times with Tyrode solution and added 1 ml of Tyrode to each well (the plates were wrapped in tin foil to protect from light). Finally imaged with Olympus IX 71 fluorescence microscope, recording luminescence intensity at 340 nm and 380 nm per second and F340/380 within 200 s (this ratio reflects intracellular basal free calcium ion levels).

Hematoxylin and eosin (H&E) staining

All vascular tissues were fixed in 10% formalin immediately after surgical resection and embedded in paraffin. Vessel tissues were cut into 5-μm sections on a microtome. The tissue sections of thoracic arteries were deparaffinated and rehydrated by immersing in xylene and gradient ethanol, then stained with hematoxylin and eosin (H&E) and examined using light microscopy. The images were obtained by Nano Zoomer slide scanner (Hamamatsu, Japan).

Artery preparation and functional assay

The procedure was performed as previously described [1]. In general, mice were sacrificed and arteries were cut into ring segments, approximately 2 mm in length. Isolated arteries were suspended in wire myograph (Danish Myo Technology, Aarhus, Denmark) for isometric tension measurement.

The implantation of HD-X10 implantable transmitter (Data Sciences International)

The procedure was performed as previously described [1]. In brief, mice were anesthetized with 1% sodium pentobarbital (i.p. injection), and the left carotid artery was isolated from the surrounding tissue. And then inserting the transmitter tube into the artery and placing HD-X10 transmitter in the flank of the mouse. Maintaining body temperature during recovering period of anesthesia. Animals were monitored until complete recovery from anesthesia. Angiotensin II (Ang II) [1000 ng/(kg·min); Cat#T8560, TargetMol, USA] was infused subcutaneously via Alzet osmotic mini pumps (#2004, Alzet, USA) as described previously. Non-invasive blood pressure was measured by tail-cuff methods (CODA-HT8 281070, Kent Scientific, USA).

Echocardiographic analysis

Male mice were anesthetized by intraperitoneal injection of sodium pentobarbital (40 mg/kg) and echocardiographic images were obtained using the Vevo 770 system (Vevo® 770 System, Visual Sonics Inc). All measurements were averaged over at least three consecutive cardiac cycles.

Pyruvate kinase activity and pyruvate production assay

Primary VSMCs or rat aorta homogenates were collected for testing pyruvate kinase activity following the descriptions of detection kit (#K709-100, Biovison, USA). The pyruvate contents were measured using detection kit (#BC2205, Solarbio, Beijing).

Xanthine oxidase activity and uric acid content assay

After treatment, VSMCs were harvested for detecting xanthine oxidase activity and uric acid content according to the instructions of the xanthine oxidase activity test kit (#AK307, Bioss, Beijing, China) and the uric acid content test kit (#AK313, Bioss, Beijing, China).

Lactate content assay

Primary VSMCs or serum samples were collected for testing lactate following the descriptions of detection kit (#KTB1100, Abbkine, China).

Total nitric oxide assay

Mice serum samples were collected for testing nitric oxide following the descriptions of Total Nitric Oxide Assay Kit (#S0024, Beyotime, China).

Glucose influx assay

After treatment, VSMCs were harvested for the glucose flux determination according to the instructions of the glucose flux detection kit (#23500, AAT Bioquest, USA). Briefly, preparing a fluorescent staining solution (2-NBDG, which is absorbed into cells through the same transport protein as glucose, and whose fluorescence intensity can effectively reflect the glucose flux of the cells) and incubating cells in a 37 ℃ incubator for 30 min. Flow cytometry (Becton Dickinson, USA) was used to detect fluorescence intensity (530/30 nm filter).

Liquid chromatography-mass spectrometry analysis of untargeted metabolomics in VSMCs

Primary VSMCs were treated with Ad-GFP/Ad-RIMKLA for 24 h before harvesting. After washing with PBS, 500 μl precooled methanol aqueous solution (volume ratio 4:1) was added into the plates and incubated at –80 ℃ for 20 min before testing (LipidALL Technologies Co., Ltd.).

Western blotting assays

Proteins were extracted from tissues or cells using Roth lysis buffer containing fresh protease and phosphatase inhibitors. Protein content in the supernatant was quantified using BCA Protein Assay Kit (#A65453, Thermo, USA). Proteins were separated by gradient SDS-PAGE and transferred to a

nitrocellulose membrane. Immunoblotting was conducted using primary antibodies against target genes. After overnight incubation with primary antibody, membranes were washed and incubated with HRP-conjugated secondary antibodies (Biodragon, China) and were detected using chemiluminescence kit (miniChemi 610, SAGECREATION, China). GAPDH was analyzed using a mouse polyclonal as loading control.

Real time PCR analysis of target mRNAs

Total RNA was extracted using the TRIzol reagent according to the manufacturer's instructions (#ET111-01, TransGen Biotech, China). Quantitative real-time PCR was performed using the DNA Engine with Chromo 4 Detector (MJ Research, Waltham, MA). The relative expressions of target genes in various groups were calculated using $2^{-\Delta\Delta Ct}$ methodology. *GAPDH* mRNA had been used as a housekeeping gene in the current study. All primer sequences used for real-time PCR assays were listed in **Additional file 1: Table S2**.

Table S1 Key materials and resources used in this study

Reagent or resource	Source	Identifier
Antibodies		
Rabbit monoclonal anti-Phospho-protein kinase B (Akt) (Ser473)	Cell Signaling Technology	Cat# 4060; RRID: AB_2315049
Rabbit polyclonal anti-Akt	Cell Signaling Technology	Cat# 9272; RRID: AB_329827
Rabbit polyclonal anti-Phospho-PKM2 (Tyr105)	Cell Signaling Technology	Cat# 3827; RRID: AB_1950369
Rabbit monoclonal anti-PKM2	Cell Signaling Technology	Cat# 4053; RRID: AB_1904096
Rabbit polyclonal anti-RIMKLA	Thermo Fisher Scientific	Cat# PA5-101852; RRID: AB_2851284
Rabbit polyclonal anti-Phospho-PTP1B (Tyr66)	Affinity Biosciences	Cat# AF8305; RRID: AB_2840367
Rabbit monoclonal anti-PTP1B	Abcam	Cat# ab244207; RRID: AB_2877148
Rabbit monoclonal anti-PCNA	ABclonal	Cat# A12427; RRID: AB_2861664
Rabbit polyclonal anti-Calponin	Proteintech	Cat# 13938-1-AP; RRID: AB_2082010
Rabbit polyclonal anti-OPN	ImmunoWay	Cat# YT3467; RRID: AB_3665642
Mouse monoclonal anti-GAPDH	OriGene	Cat# TA802563; RRID: AB_2626396
Mouse monoclonal anti-Actin, alpha-Smooth Muscle	Sigma-Aldrich	Cat# A5228; RRID:AB_262054
Alexa Fluor® 594 labeled goat anti-mouse IgG (H+L)	ZSGB-Bio	Cat# ZF-0513; RRID: AB_2892725
YSFluor™ 647 Donkey anti-rabbit lgG (H+L)	Yeasen	Cat# 34213ES60
Goat anti-mouse IgG H&L (HRP)	Biodragon	Cat# BF03001; RRID: AB_3105782
Goat Anti-Rabbit IgG H&L (HRP)	Biodragon	Cat# BF03008; RRID: AB_3073988
Bacterial and virus strains		
Adenovirus overexpression of *RIMKLA* (Ad-RIMKLA)/Ad-GFP (control)	Yan *et al.* [2]	N/A

Reagent or resource	Source	Identifier
Adeno-associated virus vector 2 with smooth muscle-specific promoter carrying *RIMKLA* gene (AAV2-SM22α-RIMKLA)/AAV-GFP (control)	Beijing Likeli Biotechnology Co., LtdS	N/A
Biological samples		
Human internal mammary artery	Xiang *et al.* [1]	N/A
Human serum samples	Beijing Chao-Yang Hospital, Capital Medical University	N/A
Chemicals, peptides, and recombinant proteins		
4',6-Diamidino-2-Phenylindole, Dihydrochloride (DAPI)	Thermo Fisher Scientific	Cat# D1306; RRID: AB_2629482
Recombinant human RIMKLA protein	Solarbio	Cat# P05051
PKM2 inhibitor (compound 3k, C3K)	Selleck	Cat# S8616
ATP-P2 receptor inhibitor (Suramin)	Abcam	Cat# ab120422
PTP1B inhibitor (PTP1B-IN-2)	Selleck	Cat# S0119
ROS scavenger (NAC)	Beyotime	Cat# ST1546
Xanthine oxidase inhibitor (Febuxostat, FB)	Beyotime	Cat# SF1114
Angiotensin II human acetate	TargetMol	Cat# T8560
Methylthiazolyldiphenyl-tetrazolium bromide (MTT)	Beyotime	Cat# ST316
RNase A	Sigma	Cat# R4642
Fura-2 AM	Invitrogen	Cat# F1221
Tetramethylrhodamine methyl ester (TMRM)	Invitrogen	Cat# M20036
2′,7′-Dichlorodihydrofluorescein diacetate (DCFH-DA)	Invitrogen	Cat# C400
Phalloidin	Yeasen	Cat# 40734ES75
Glutathione sepharose 4B resin	Solarbio	Cat# P2020

Reagent or resource	Source	Identifier
Critical commercial assays		
transfection kit (BioShuttle siRNA/miRNA)	Biodragon	Cat# KX0110049
ATP-Lite Assay Kit	Vigorous Biotechnology	Cat# T007
Pyruvate kinase activity detection kit	Biovison	Cat# K709-100
Pyruvate content detection kit	Solarbio	Cat# BC2205
Xanthine oxidase activity assay kit	Bioss	Cat# AK307
Uric acid content test kit	Bioss	Cat# AK313
Glucose flux detection kit	AAT Bioquest	Cat# 23500
Pierce immunoprecipitation kit	Thermo Fisher Scientific	Cat# 26147
GST-tag Protein Purification Kit	Beyotime	Cat# P2262
Total RNA Extraction Kit	TransGen Biotech	Cat# ET111-01
BCA Protein Assay Kit	Thermo Fisher Scientific	Cat# A65453
Mitochondrial oxidative phosphorylation (OXPHOS) assay kits	Alicelligent Technologies	Cat# ALS22012
Total Nitric Oxide Assay Kit	Beyotime	Cat# S0024
Deposited data		
The PDB formats of PKM2 (6JFB) and PTP1B (1A5Y) structure	Protein Data Bank PDB database	http://www.rcsb.org/
The AlphaFold prediction of RIMKLA (AF-Q8IXN7-F1)	AlphaFold	https://alphafold.com/
Conserved *RIMKLA* gene sequence	UCSC	http://genome.ucsc.edu/
GWAS catalog for *RIMKLA* gene	GWAS	https://www.ebi.ac.uk/gwas/
Experimental models: cell lines		

Reagent or resource	Source	Identifier
Rat primary vascular smooth muscle cell	Xiang *et al*. [1]	N/A
Mouse primary vascular smooth muscle cell	Xiang *et al*. [1]	N/A
Experimental models: organisms/strains		
Mouse: C57BL/6	The department of laboratory science of Peking University Health Science Center	N/A
Rat: spontaneously hypertensive rat (SHR)	The department of laboratory science of Peking University Health Science Center	N/A
Rat: Sprague-Dawley (SD)	The department of laboratory science of Peking University Health Science Center	N/A
Rat: Salt-sensitive hypertensive Dahl/SS	Beijing Vital River Laboratory Animal Technology Co., Ltd.	N/A
Rat: SS-13^{BN} (control rat of salt-sensitive hypertensive Dahl/SS)	Beijing Vital River Laboratory Animal Technology Co., Ltd.	N/A
Mouse: PKM2$^{flox/flox}$	Professor Juan Feng (Peking University Health Science Center)	N/A
Mouse: Tagln-Cre	Xiang *et al.* [1]	N/A
Mouse: RIMKLA$^{flox/flox}$	Yan *et al.* [2]	N/A
Oligonucleotides		
The sequence of siRIMKLA:<br>forward 5’-AAGGAAGGUUCUAGAAAUA-3’;<br>reverse 5’-UAUUUCUAGAACCUUCCUU-3’	This paper	N/A
Primers for RT-PCR, see **Additional file 1: Table S7**	This paper	N/A
Recombinant DNA		

Reagent or resource	Source	Identifier
pAAV-mSM22a promoter-mRimkla-3flag-ZsGreen	This paper	N/A
pGEX-4t-1-GST-PKM2	This paper	N/A
Software and algorithms		
GraphPad Prism 10.4.1	GraphPad Software Inc.	https://www.graphpad.com/
Vevo® 770 System	Visual Sonics Inc.	https://www.visualsonics.com/
Protein docking program (ZDOCK)	Angerani *et al*. [3]	http://zdock.umassmed.edu/
PyMOL	https://pymol.org/2/	N/A
Wave 2.6.3 software	Alicelligent Technologies, Beijing, China	N/A
ImageJ 1.53e	https://imagej.net/ij/	N/A
Other		
Alzet osmotic mini pumps	Alzet (USA)	Cat# 2004
HD-X10 transmitter	Data Sciences International, DSI (Harvard Bioscience, Inc.)	https://www.datasci.com/
Wire myograph for vascular reactivity	Danish Myo Technology (Denmark)	https://www.dmt.dk/
BD LSR Fortessa flow cytometer	Becton Dickinson	https://www.bd.com/
Confocal laser scanning microscope (TCS-SP8 STED 3X)	Leica (Germany)	https://cn.leica-microsystems.com.cn/
Nano Zoomer slide scanner	Hamamatsu (Japan)	https://www.hamamatsu.com.cn/
Varioskan™ LUX multimode microplate reader	Thermo Fisher Scientific (USA)	https://www.thermofisher.cn/
Chemiluminescence meter (miniChemi 610)	SAGECREATION (China)	http://www.sagecreation.com.cn/
UV Transilluminator (ChampUV)	SAGECREATION (China)	http://www.sagecreation.com.cn/

Reagent or resource	Source	Identifier
Seahorse XFe24 Extracellular Flux Analyzer	Agilent Technologies (California, USA)	https://www.agilent.com.cn/
Transwell plate	Corning (USA)	Cat# CLS3422
Olympus Ix 71 fluorescence microscope	Olympus (Japan)	https://www.olympus.com.cn/
DNA engine with chromo 4 detector	MJ Research, Inc. (Waltham, MA)	N/A

Akt. Protein kinase B; PKM2. M2-type pyruvate kinase; RIMKLA. Ribosomal modification protein rimK-like family member A; PTP1B. Protein-tyrosine phosphatase 1B; PCNA. Proliferating cell nuclear antigen; OPN. Osteopontin; GAPDH. Glyceraldehyde-3-phosphate dehydrogenase; NAC. N-acetyl-L-cysteine; ATP. Adenosine triphosphate; GST. Glutathione S-transferase; GWAS. Genome-wide association studies; Ad-RIMKLA. Adenoviral RIMKLA; AAV2-SM22α-RIMKLA. Adeno-associated virus vector 2 with smooth muscle specific promoter carrying *RIMKLA* gene

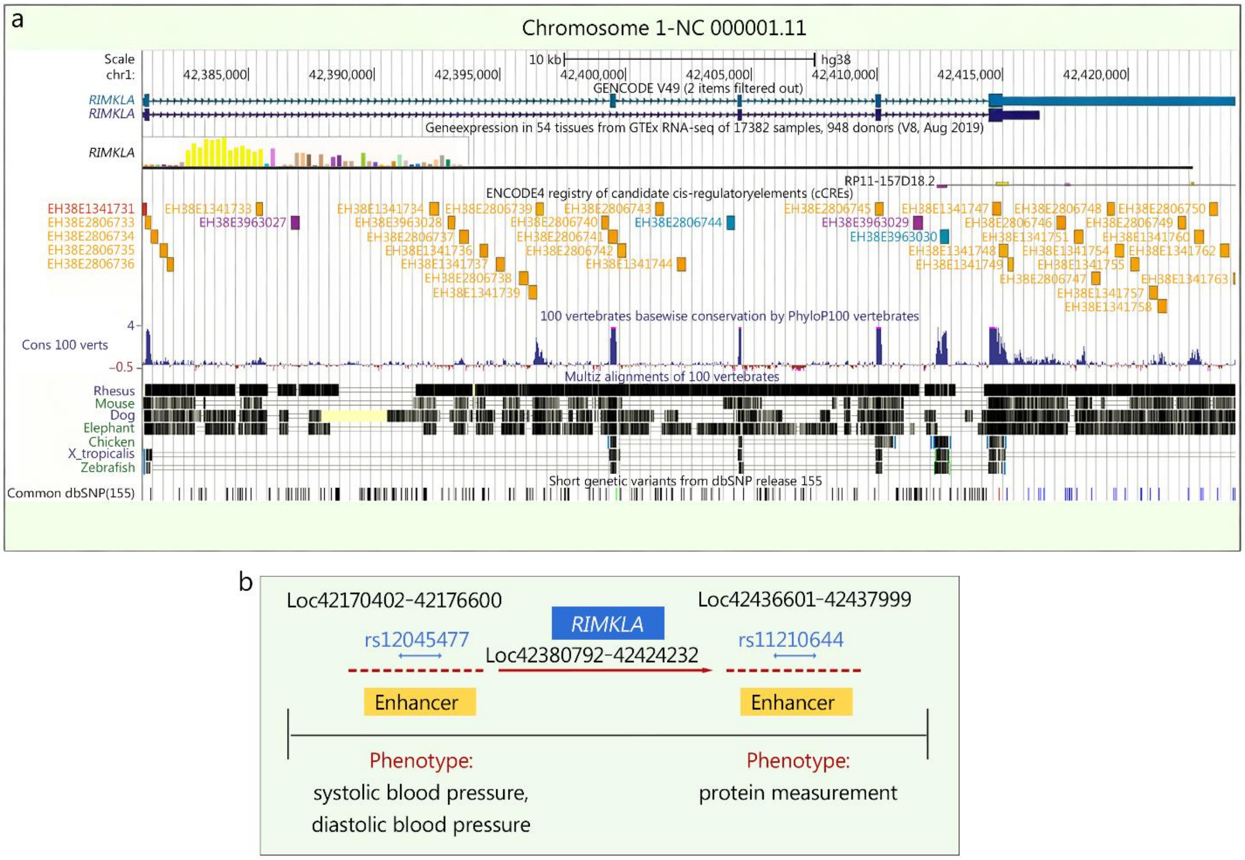

Fig. S1 *RIMKLA* gene is highly conserved and associated with the regulation of blood pressure. **a** Highly genetical conservation of *RIMKLA* gene between human and mouse is shown (http://genome.ucsc.edu). **b** Phenotypes from GWAS catalog for *RIMKLA* gene in blood pressure regulation (https://www.ebi.ac.uk/gwas). RIMKLA. Ribosomal modification protein rimK-like family member A; GWAS. Genome-wide association studies

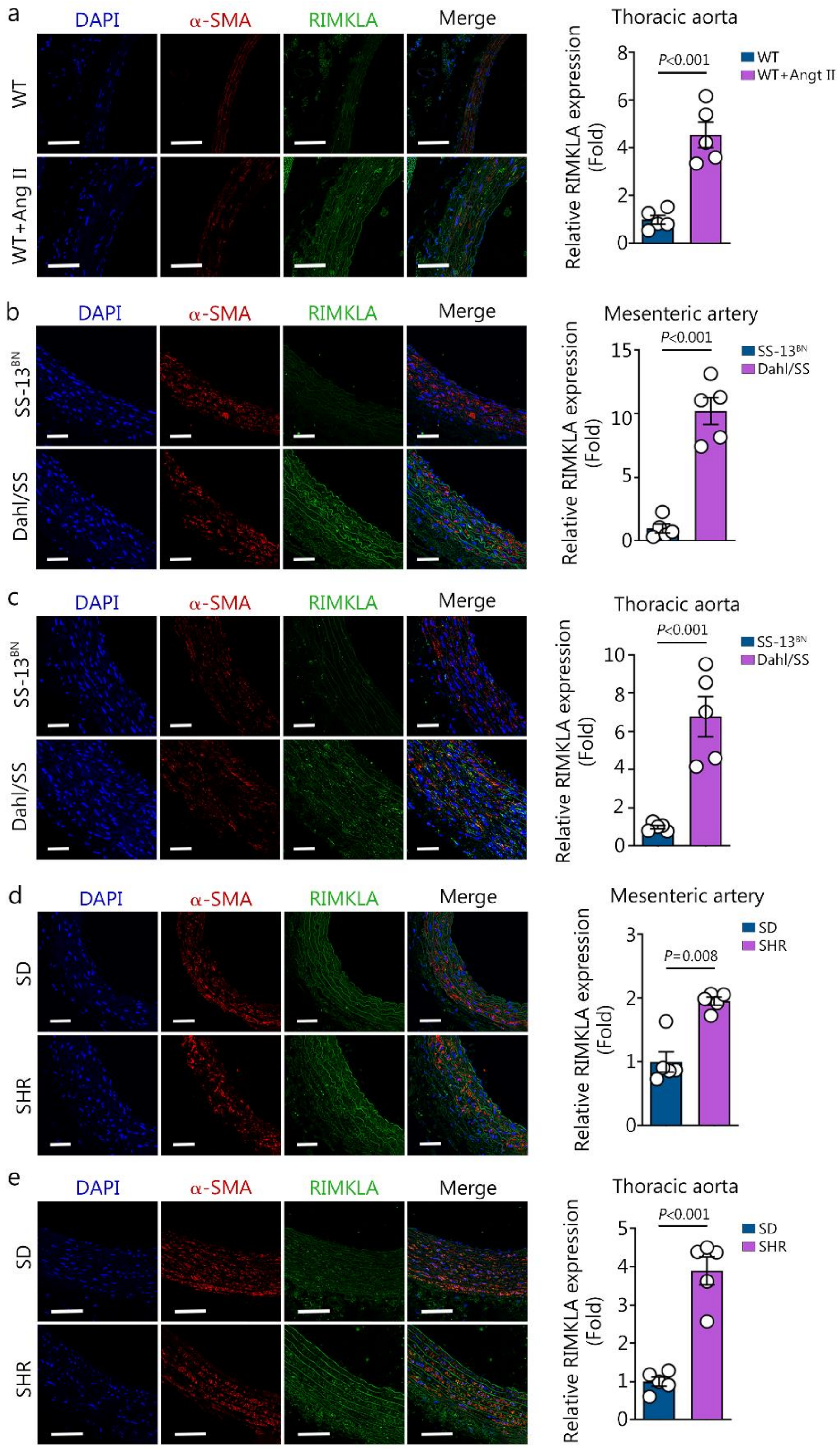

Fig. S2 RIMKLA protein level is increased in the arteries of hypertensive animals. **a** Representative images of immunofluorescent staining of RIMKLA protein in the thoracic artery of Ang II-induced hypertensive mice and the quantitative data analysis (*n*=5). Scale bar=75 μm. Representative images of immunofluorescent staining of RIMKLA protein in mesenteric (**b**) and thoracic artery (**c**) of salt-sensitive

hypertensive Dahl/SS rats, and the quantitative data analysis (*n*=5). Scale bar=50 μm. Representative images of immunofluorescent staining of RIMKLA protein in mesenteric (**d**) and thoracic artery (**e**) of SHR rats, and the quantitative data analysis (*n*=5). Scale bar=50 μm for (**d**) and 75 μm for (**e**). *P*-value for (**d**) is analyzed by Mann-Whitney *U* test, others are determined by unpaired Student's *t*-test. RIMKLA. Ribosomal modification protein rimK-like family member A; DAPI. 4,6'-diamidino-2-phenylindole; α-SMA. α-smooth muscle actin; WT. Wild type; Ang II. Angiotensin II; SD. Sprague-Dawley; SHR. Spontaneously hypertensive rat

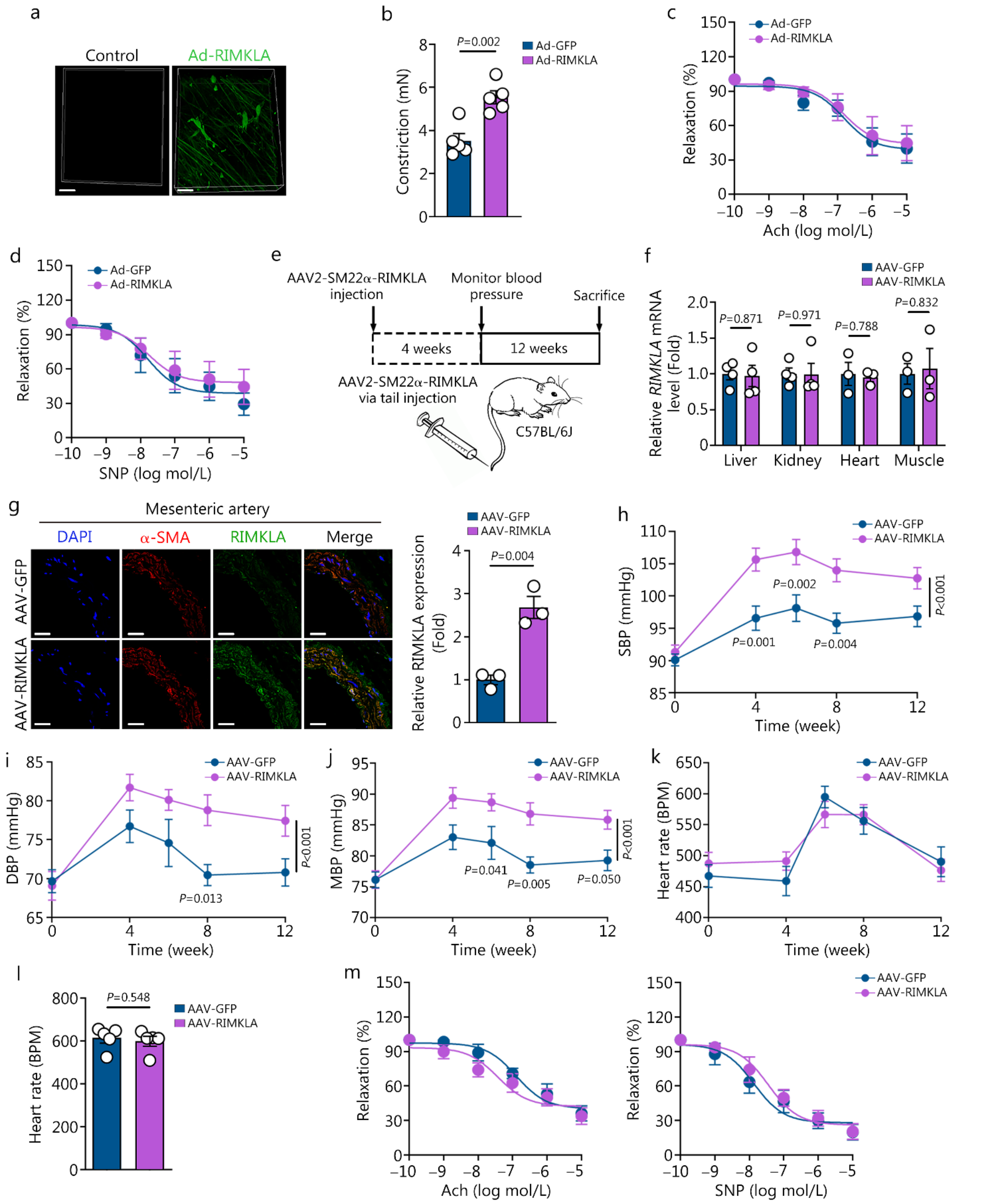

Fig. S3 VSMC-specific overexpression of *RIMKLA* increases vessel contractility and blood pressure in mice. **a** Representative 3D images of preincubation with Ad-RIMKLA (with GFP tag) for 12 h in the mesenteric artery of SD rats. Scale bar=75,000 mm. **b** Mesenteric arteries of SD rats preincubated with

Ad-RIMKLA had stronger contractive responses to potassium than those of control arteries treated with Ad-GFP (*n*=5). Mesenteric artery of SD rats preincubated with Ad-RIMKLA had comparable endothelial-dependent (**c**) and endothelial-independent relaxation (**d**) in response to Ach and SNP as that treated with Ad-GFP (*n*=5). **e** The schematic procedure for obtaining VSMC-specific overexpression of RIMKLA in mice. Eight to ten weeks old male C57BL/6J mice were injected with AAV-RIMKLA or AAV2-GFP (5×10^{11} vg/per mouse) via tail vein. After injection for one month, the blood pressure and heart rate of mice were measured by the tail-cuff method. **f** *RIMKLA* gene mRNA level was similar in the liver, kidney, heart, and muscle of mice injected with AAV-RIMKLA or AAV-GFP (*n*=3–4). **g** Immunofluorescence confocal microscopy revealed that RIMKLA protein level was increased in the mesenteric artery of AAV-RIMKLA-injected mice when compared with AAV-GFP-injected mice. The representative images were shown in the left panel, and quantitative data were shown in the right (*n*=3). Scale bar=25 μm. In 4, 6, 8 and 12 weeks after injection, C57BL/6J mice injected with AAV-RIMKLA had higher SBP and MBP than the control mice injected with AAV-GFP (**h, j**), while the DBP of AAV-RIMKLA-injected mice was significantly higher than AAV-GFP injected mice at 8 and 12 weeks after injection (**i**), with no statistical difference in heart rates between the two groups (**k**) (*n*=10). **l** The mice injected with AAV-RIMKLA or AAV-GFP had comparable heart rate (*n*=5). **m** Thoracic arteries of AAV-RIMKLA- and AAV-GFP-injected mice had similar Ach-induced and SNP-induced dilation (*n*=4). *P*-values for (**b, f, g, l**) are determined by unpaired *t*-test, and others are determined by two-way ANOVA followed by Bonferroni correction and within-test multiple corrections using Tukey's multiple analysis. VSMC. Vascular smooth muscle cell; RIMKLA. Ribosomal modification protein rimK-like family member A; Ad-RIMKLA. Adenoviral RIMKLA; AAV. Adeno-associated virus; GFP. Green fluorescent protein; AAV2-RIMKLA. Adeno-associated virus vector 2 with smooth muscle specific promoter carrying *RIMKLA* gene; DAPI. 4,6'-diamidino-2-phenylindole; α-SMA. α-smooth muscle actin; Ach. Acetylcholine; SNP. Sodium nitroprusside dehydrate; SBP. Systolic blood pressure; DBP. Diastolic blood pressure; MBP. Mean blood pressure

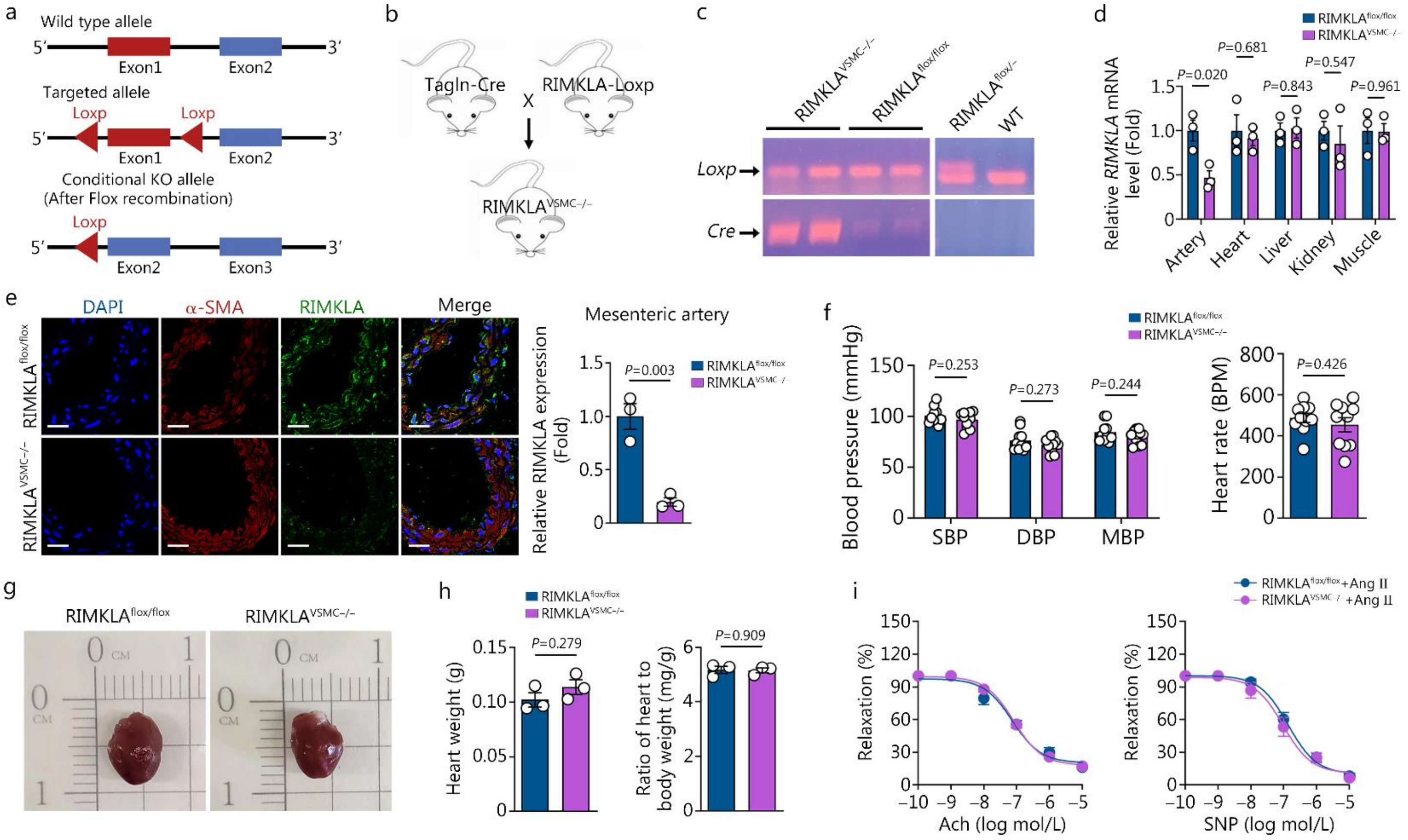

Fig. S4 Generation and characterization of VSMC-specific deletion of *RIMKLA* in mice. **a** RIMKLA-Loxp mice were generated by inserting two Loxp sites on both sides of exon1 in mouse RIMKLA gene. **b** RIMKLA-Loxp mice were crossed with Tagln-Cre mice to generate RIMKLA$^{VSMC-/-}$ mice. **c** DNA agarose gel electrophoresis exhibited that both *Loxp* and *Cre* genes were expressed in RIMKLA$^{VSMC-/-}$ mice, while RIMKLA$^{flox/flox}$ mice only expressed *Loxp* gene. **d** *RIMKLA* gene mRNA level was reduced in the mesenteric artery, but not in the heart, liver, kidney, and muscle of RIMKLA$^{VSMC-/-}$ mice (*n*=3). **e** Immunofluorescence microscopy certified that RIMKLA protein level was significantly decreased in the mesenteric artery of RIMKLA$^{VSMC-/-}$ mice when compared with RIMKLA$^{flox/flox}$ mice (*n*=3). Scale bar=25 μm. **f** RIMKLA$^{VSMC-/-}$ mice (8–10-week-old) had comparable blood pressure and heart rates to RIMKLA$^{flox/flox}$ mice in physiological condition (*n*=10). No significant change in cardiac morphology (**g**), heart weight and heart to body weight ratio (**h**) in RIMKLA$^{VSMC-/-}$ mice when compared with RIMKLA$^{flox/flox}$ mice (*n*=3). **i** Artery tension measurement revealed that thoracic arteries of VSMC-specific deletion of *RIMKLA* had no effect on Ach-induced and SNP-induced relaxation after Ang II treatment (*n*=3). Arteries were preincubated with 100 nmol/L Ang II for 10 min before vascular tone was determined. *P*-values for (**i**) is determined by two-way ANOVA followed by Bonferroni correction and within-test multiple corrections using Tukey's multiple analysis. Others are analyzed by unpaired *t*-test.

VSMC. Vascular smooth muscle cell; RIMKLA. Ribosomal modification protein rimK-like family member A; KO. Knockout; Ad-RIMKLA. Adenoviral RIMKLA; DAPI. 4,6'-diamidino-2-phenylindole; α-SMA. α-smooth muscle actin; Ach. Acetylcholine; SNP. Sodium nitroprusside dehydrate; Ang II. Angiotensin II

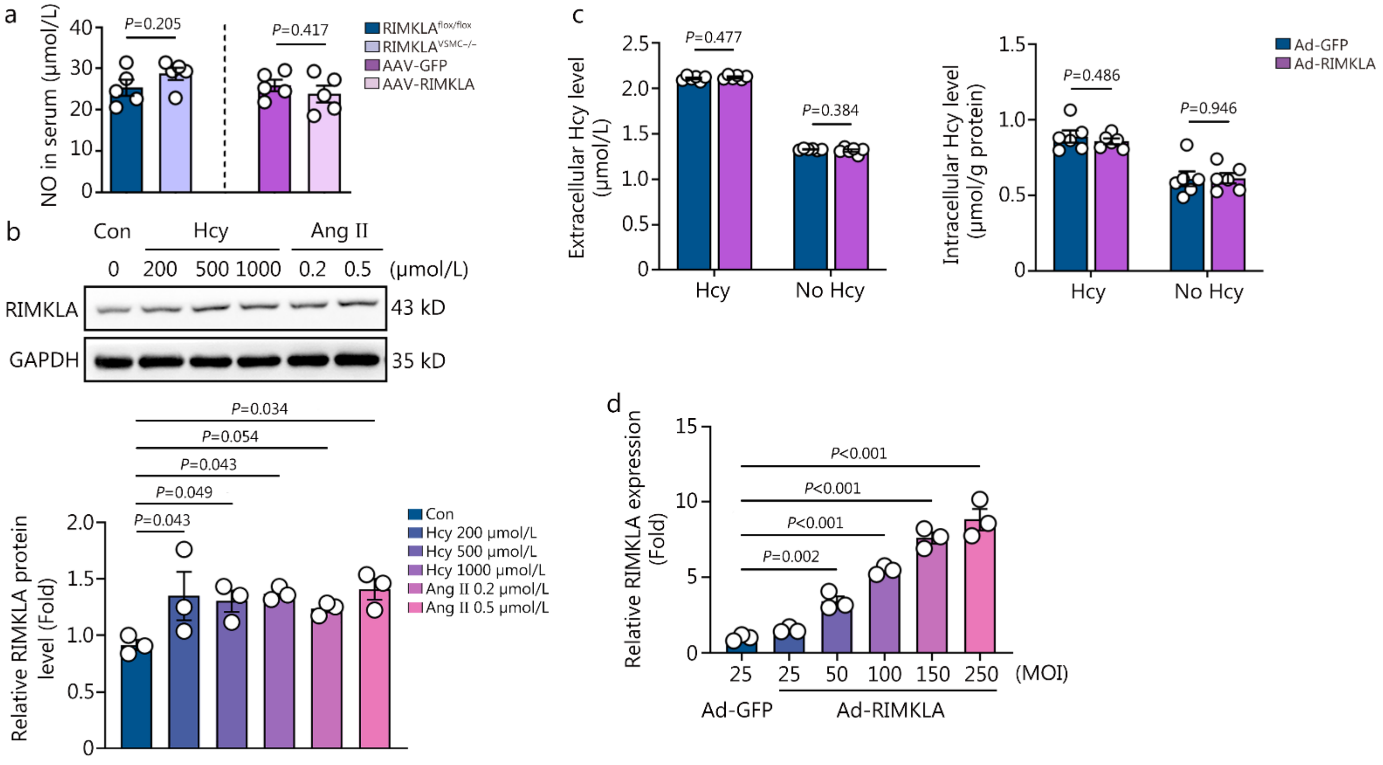

Fig. S5 RIMKLA is induced by two risk factors (Hcy and Ang II) in VSMCs. **a** Serum nitric oxide levels were not different between RIMKLA$^{VSMC-/-}$ and RIMKLA$^{flox/flox}$ mice, as well as between AAV-RIMKLA-injected mice and AAV-GFP-injected mice (*n*=5). **b** Hcy and Ang II upregulated RIMKLA expression in VSMCs (*n*=3). **c** *RIMKLA* overexpression in VSMCs had little effect on extracellular and intracellular Hcy levels, with or without Hcy treatment (500 μmol/L) (*n*=6). **d** Treatment with adenovirus RIMKLA (Ad-RIMKLA) dose-dependently increased RIMKLA protein level. Quantitative data shown in the figure. Primary rat VSMCs were treated with different doses of Ad-RIMKLA for 24 h. Ad-GFP (25 MOI) is used as control (*n*=3). *P*-value for (**b, d**) are determined by one-way ANOVA followed by Bonferroni post hoc analysis. Others are determined by unpaired Student's *t*-test. VSMC. Vascular smooth muscle cell; RIMKLA. Ribosomal modification protein rimK-like family member A; AAV. Adeno-associated virus; GFP. Green fluorescent protein; Ang II. Angiotensin II; Ad-RIMKLA. Adenoviral RIMKLA; Hcy. Homocysteine; MOI. Multiplicity of infection

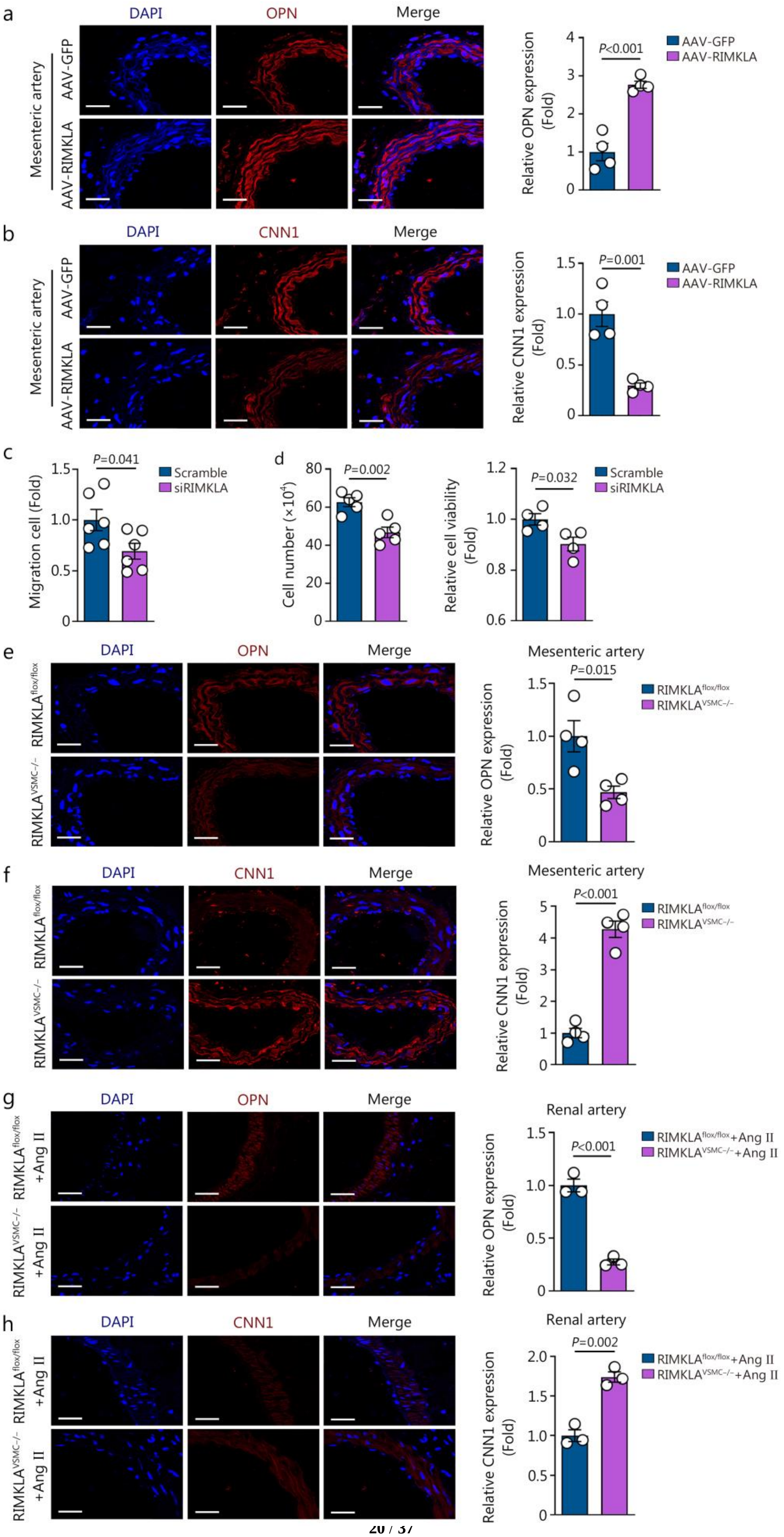

a
DAPI
OPN
Merge
Mesenteric artery
AAV-GFP
AAV-RIMKLA
Relative OPN expression (Fold)
P<0.001
AAV-GFP
AAV-RIMKLA
b
DAPI
CNN1
Merge
Mesenteric artery
AAV-GFP
AAV-RIMKLA
Relative CNN1 expression (Fold)
P=0.001
AAV-GFP
AAV-RIMKLA
c
Migration cell (Fold)
P=0.041
Scramble
siRIMKLA
d
Cell number (×10^4)
P=0.002
Relative cell viability (Fold)
P=0.032
Scramble
siRIMKLA
e
DAPI
OPN
Merge
RIMKLA^flox/flox
RIMKLA^VSMC−/−
Mesenteric artery
P=0.015
Relative OPN expression (Fold)
RIMKLA^flox/flox
RIMKLA^VSMC−/−
f
DAPI
CNN1
Merge
RIMKLA^flox/flox
RIMKLA^VSMC−/−
Mesenteric artery
P<0.001
Relative CNN1 expression (Fold)
RIMKLA^flox/flox
RIMKLA^VSMC−/−
g
DAPI
OPN
Merge
RIMKLA^flox/flox +Ang II
RIMKLA^VSMC−/− +Ang II
Renal artery
P<0.001
Relative OPN expression (Fold)
RIMKLA^flox/flox+Ang II
RIMKLA^VSMC−/−+Ang II
h
DAPI
CNN1
Merge
RIMKLA^flox/flox +Ang II
RIMKLA^VSMC−/− +Ang II
Renal artery
P=0.002
Relative CNN1 expression (Fold)
RIMKLA^flox/flox+Ang II
RIMKLA^VSMC−/−+Ang II

Fig. S6 RIMKLA promotes VSMC phenotype switch from contractile to synthetic in animal models. Representative images of immunofluorescent staining of OPN (**a**) and CNN1 proteins (**b**) in the mesenteric artery of AAV-RIMKLA and AAV-GFP mice (*n*=4). Scale bar=25 μm. **c** *RIMKLA* knockdown restrained the migration of VSMCs as assessed by Transwell assay (*n*=6). **d** *RIMKLA* knockdown inhibited the proliferation of VSMCs as evaluated by cell number counting and cell viability assays (*n*=4–5). Primary rat VSMCs were transfected with 50 nmol/L siRIMKLA for 24 h before experiments. Representative immunofluorescent staining images of OPN protein (**e**) and CNN1 protein (**f**) in the mesenteric artery of $RIMKLA^{VSMC-/-}$ mice and $RIMKLA^{flox/flox}$ mice (*n*=4). Scale bar=25 μm. Representative images of OPN expression (**g**) and CNN1 expression (**h**) in the renal artery of Ang II-induced hypertensive $RIMKLA^{VSMC-/-}$ mice and control mice (*n*=3). Scale bar=25 μm. All *P*-values are determined by unpaired Student's *t*-test. VSMC. Vascular smooth muscle cell; OPN. Osteopontin; CNN1. Calponin 1; RIMKLA. Ribosomal modification protein rimK-like family member A; Ad-RIMKLA. Adenoviral RIMKLA; AAV. Adeno-associated virus; GFP. Green fluorescent protein; Ang II. Angiotensin II

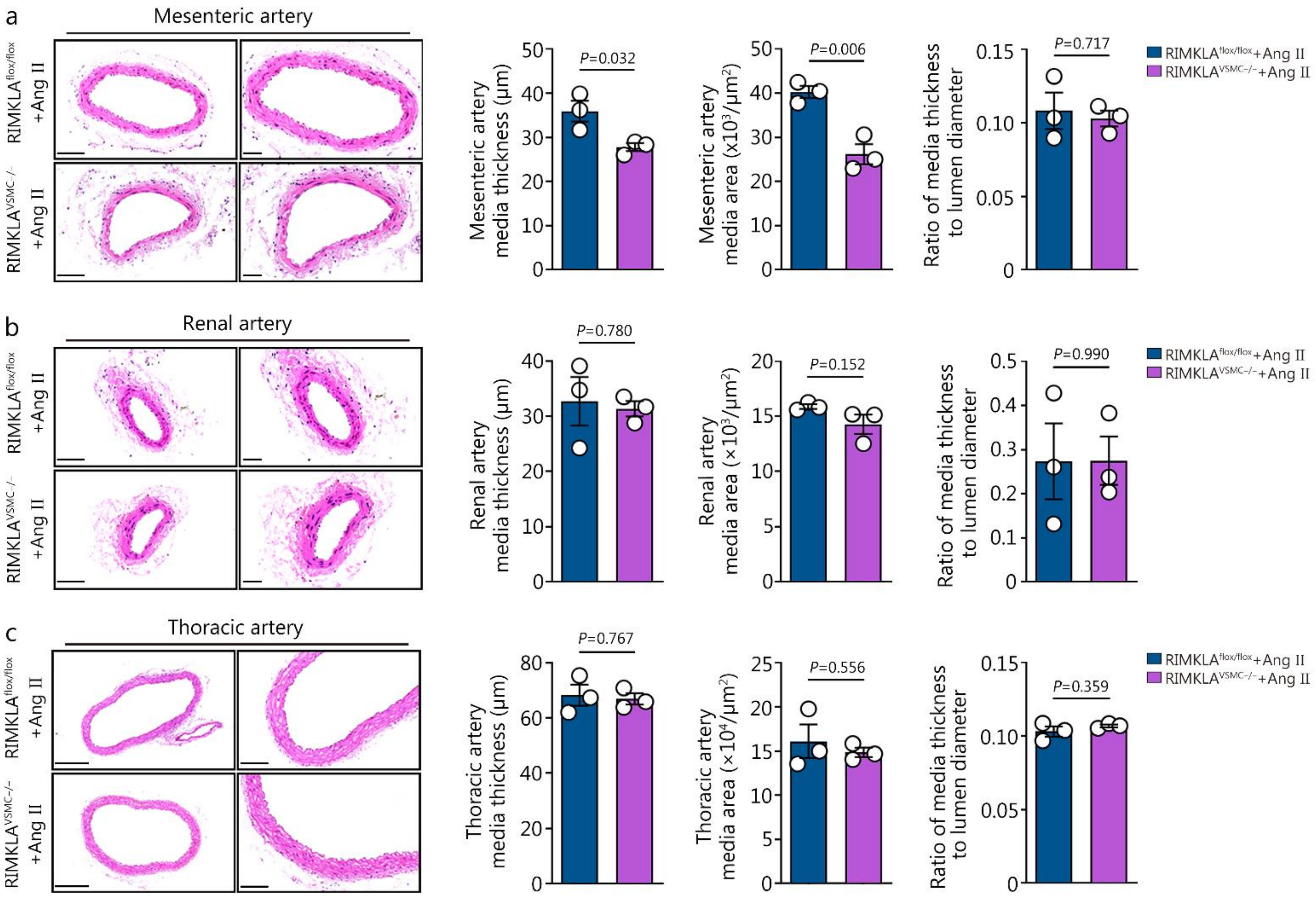

Fig. S7 VSMC-specific deletion of *RIMKLA* attenuates Ang II-induced artery remodeling. **a** Representative H&E staining images of mesenteric artery of RIMKLA$^{VSMC-/-}$ mice and RIMKLA$^{flox/flox}$ mice after treating with Ang II [1000 ng/(kg·min)] for one month. Scale bar=100 μm (left) and 50 μm (right). Quantitative data analysis of mesenteric media thickness, media area, and ratio of media thickness to lumen diameter (*n*=3). **b** Representative H&E staining images of renal artery of RIMKLA$^{VSMC-/-}$ mice and RIMKLA$^{flox/flox}$ mice after treating with Ang II [1000 ng/(kg·min)] for one month. Scale bar=100 μm (left) and 50 μm (right). Quantitative data analysis of renal media thickness, media area, and ratio of media thickness to lumen diameter (*n*=3). **c** Representative H&E staining images of the thoracic artery of RIMKLA$^{VSMC-/-}$ mice and RIMKLA$^{flox/flox}$ mice after treating with Ang II [1000 ng/(kg·min)] for one month. Scale bar=250 μm (left) and 100 μm (right). Quantitative data analysis of thoracic media thickness, media area, and ratio of media thickness to lumen diameter (*n*=3). All *P*-values are determined by unpaired Student's *t*-test. VSMC. Vascular smooth muscle cell; RIMKLA. Ribosomal modification protein rimK-like family member A; Ang II. Angiotensin II

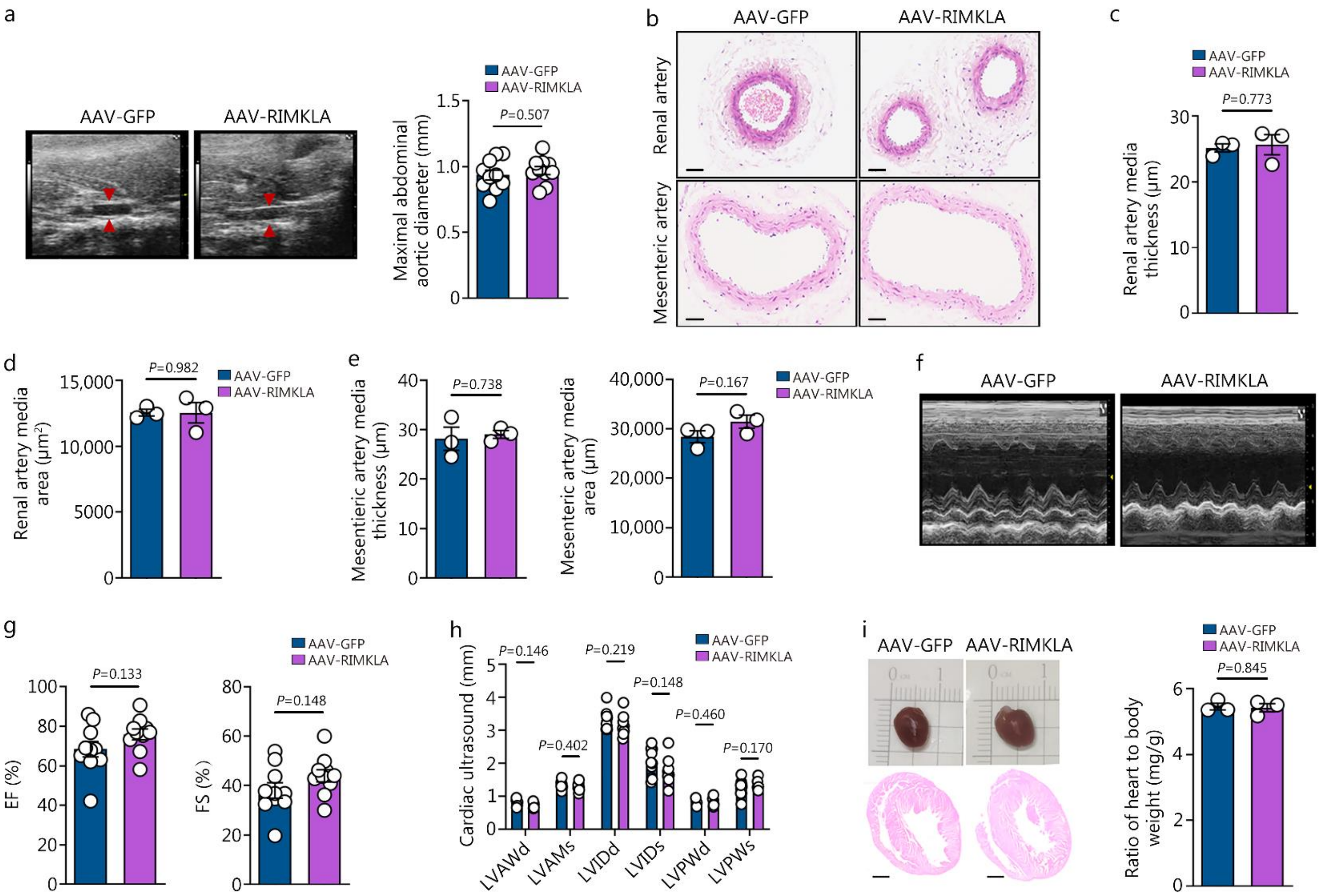

Fig. S8 VSMC-specific overexpression of *RIMKLA* has little effect on cardiovascular remodeling. **a** Abdominal aorta echocardiography exhibited no difference in the maximal abdominal aorta diameters between AAV-RIMKLA-injected and AAV-GFP-injected mice (*n*=10). **b** Representative H&E staining of renal and mesenteric arteries in AAV-RIMKLA-injected and AAV-GFP-injected mice. Scale bar=50 μm. VSMC-specific overexpression of *RIMKLA* presented no difference in the media layer thickness and area of renal arteries (**c, d**) and mesenteric arteries (**e**) in mice (*n*=3). **f** Representative images of cardiac ultrasonography in the AAV-RIMKLA-injected and AAV-GFP-injected mice. **g** AAV-RIMKLA-injected mice had comparable left ventricular ejection fraction (EF) and fractional shortening (FS) with AAV-GFP-injected mice (*n*=10). **h** Quantitative data of cardiac ultrasonography analysis (*n*=10). **i** Cardiac morphology and heart to body weight ratio exhibited no significant changes between AAV-RIMKLA-injected and AAV-GFP-injected mice. Scale bar=1 mm. All *P*-values are determined by unpaired Student's *t*-test. VSMC. Vascular smooth muscle cell; RIMKLA. Ribosomal modification protein rimK-like family member A; AAV. Adeno-associated virus; GFP. Green fluorescent protein; LVAWd. Left ventricular anterior wall thickness at end-diastole; LVAWs. Left ventricular anterior wall thickness at end-

systole; LVIDd. Left ventricular internal dimension at end-diastole; LVIDs. Left ventricular internal dimension at end-systole; LVPWd. Left ventricular posterior wall thickness at end-diastole; LVPWs. Left ventricular posterior wall thickness at end-systole

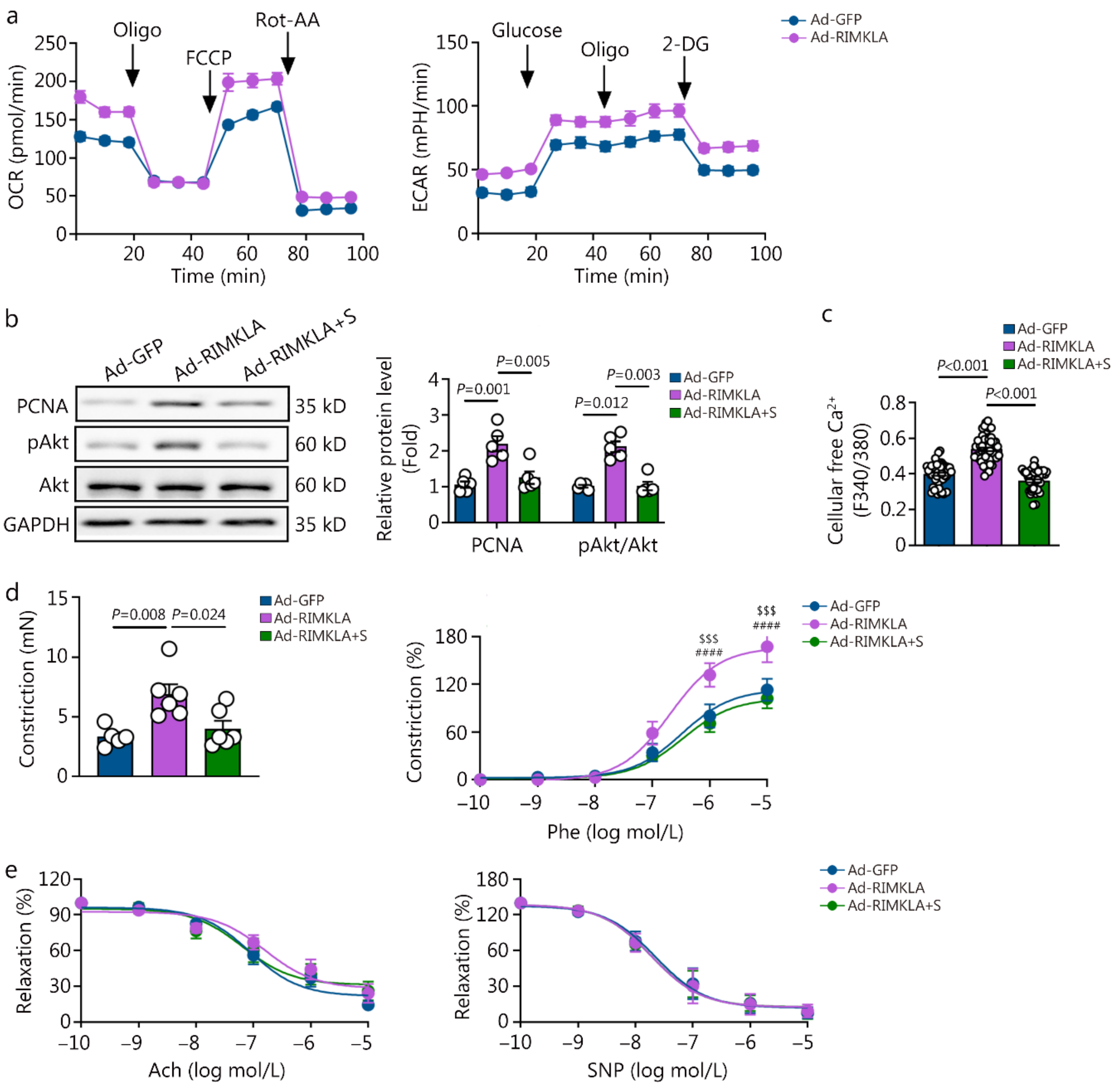

Fig. S9 Treatment of P2 receptor inhibitor reverses RIMKLA-induced vasoconstriction. **a** *RIMKLA* overexpression increased the oxygen consumption rate (OCR) and extracellular acidification rate (ECAR) in VSMCs (*n*=6). OCR and ECAR were measured by the Seahorse XFe24 Extracellular Flux Analyzer (Agilent Technologies, California, USA). Oligomycin (Oligo; 1.5 μmol/L), carbonylcyanide-4-(trifluoromethoxy)-phenylhydrazone (FCCP; 1.0 μmol/L), and rotenone plus antimycin A (Rot-AA; 1.0 μmol/L) solutions were sequentially injected into the wells of the culture microplate for OCR analysis. Glucose (10 mmol/L), Oligo (1.5 μmol/L), and 2-deoxy-D-glucose (2-DG; 50 mmol/L) solutions were sequentially injected for ECAR analysis. **b** Suramin (S) suppressed the increase of PCNA expression and Akt activation elicited by overexpression of *RIMKLA* (*n*=5). Rat VSMCs were treated with Ad-RIMKLA in the absence or presence of 40 μmol/L P2 receptor inhibitor suramin for 24 h. **c** Suramin blocked the increase in cellular Ca^{2+} level stimulated by overexpression of *RIMKLA*. Data were obtained from at least

50 cells from at least 3 independent experiments. Suramin restrained the increased vasoconstriction induced by overexpression of *RIMKLA* (**d**), with no effect on endothelial-dependent and -independent relaxation (**e**) of SD arteries (*n*=6). *P*-values were determined by one-way ANOVA with Bonferroni post hoc (**b**, **d**-left) and Kruskal-Wallis test with Dunn's analysis (**c**). Others are analyzed by two-way ANOVA with Bonferroni and Tukey's corrections. $^{\$\$\$}P<0.001$, Ad-RIMKLA vs. Ad-GFP; $^{\#\#\#\#}P<0.0001$, Ad-RIMKLA+S vs. Ad-RIMKLA. VSMC. Vascular smooth muscle cell; RIMKLA. Ribosomal modification protein rimK-like family member A; Ad-RIMKLA. Adenoviral RIMKLA; GFP. Green fluorescent protein; PCNA. Proliferating cell nuclear antigen; Akt. Protein kinase B; Ach. Acetylcholine; SNP. Sodium nitroprusside dehydrate

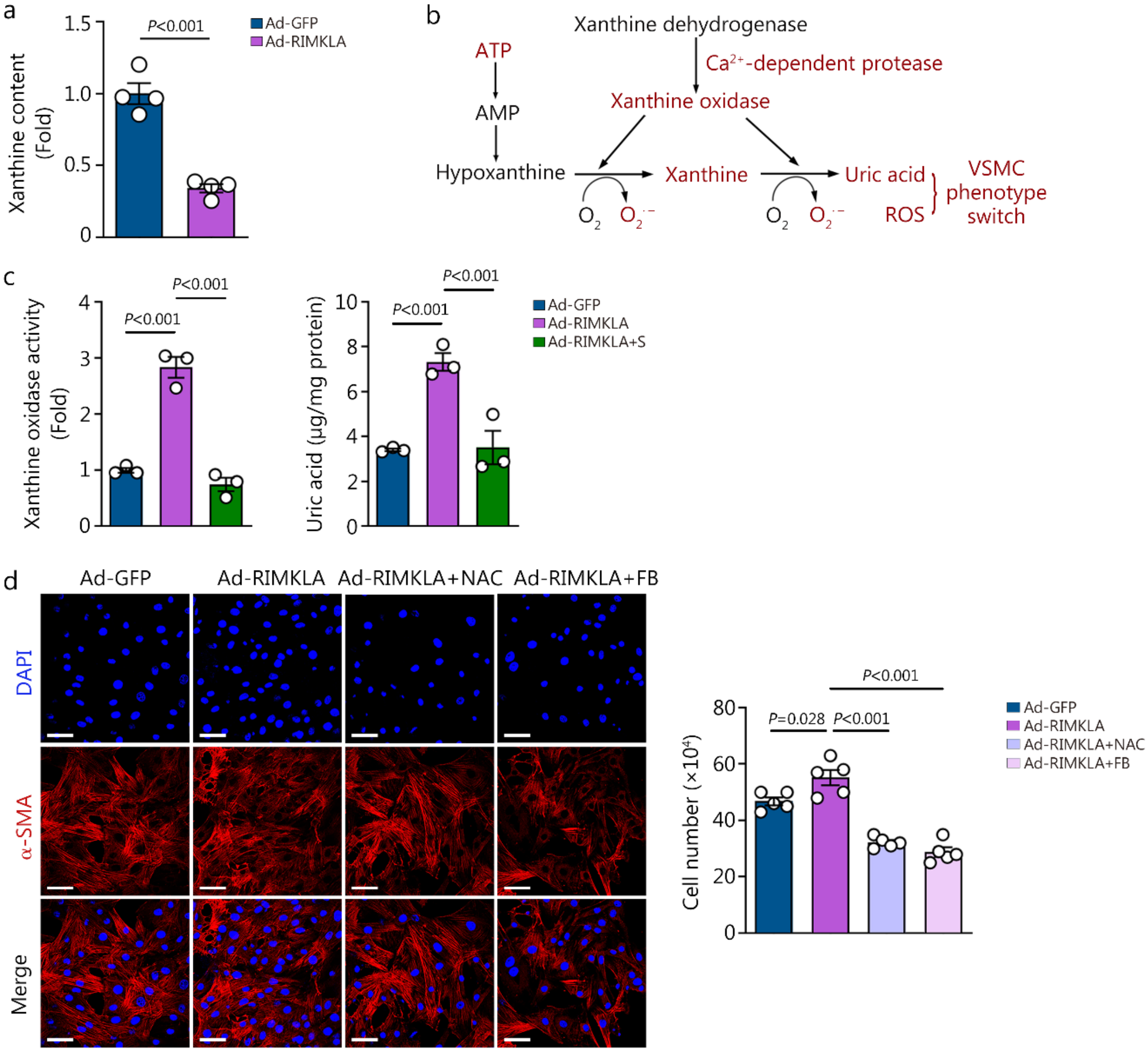

Fig. S10 ROS and uric acid are involved in RIMKLA-induced VSMC phenotype switch. **a** Metabolomic analysis revealed that xanthine was significantly decreased after overexpression of *RIMKLA* in VSMCs (*n*=4). **b** The schematic graph of ATP metabolism. **c** Treatment of P2 receptor inhibitor suppressed the augment in xanthine oxidase activity and uric acid production caused by overexpression of *RIMKLA* (*n*=4–6). Rat VSMCs were treated with Ad-GFP or Ad-RIMKLA with or without suramin (40 μmol/L) for 24 h. **d** ROS scavenger (NAC, 10 μmol/L) and xanthine oxidase inhibitor (FB, 10 μmol/L) inhibited RIMKLA-promoted proliferation of VSMCs, as evaluated by cell confocal images and cell number counting assay (*n*=5). Scale bar=50 μm. *P*-values are determined by one-way ANOVA with Bonferroni post hoc (**c, d**), and others are analyzed by unpaired *t*-test. NAC. N-Acetyl-L-cysteine; FB. Febuxostat; VSMC. Vascular smooth muscle cell; RIMKLA. Ribosomal modification protein rimK-like family member A; Ad-RIMKLA. Adenoviral RIMKLA; GFP. Green fluorescent protein; ATP. Adenosine triphosphate; AMP. Adenosine monophosphate; ROS. Reactive oxygen species; DAPI. 4,6'-diamidino-2-phenylindole; α-SMA. α-smooth muscle actin

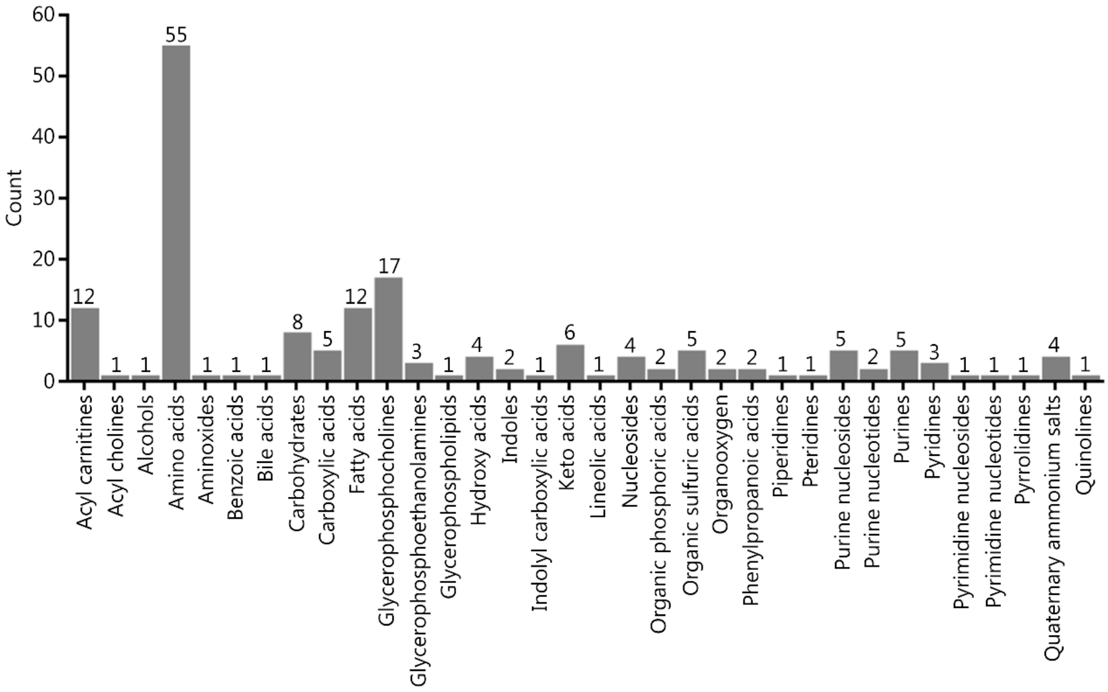

Fig. S11 Metabolome summary of metabolites in VSMCs after *RIMKLA* overexpression (*n*=4). Rat primary VSMCs were infected with Ad-RIMKLA or Ad-GFP for 24 h before metabolomic analysis was performed. VSMC. Vascular smooth muscle cell; RIMKLA. Ribosomal modification protein rimK-like family member A; Ad-RIMKLA. Adenoviral RIMKLA; GFP. Green fluorescent protein

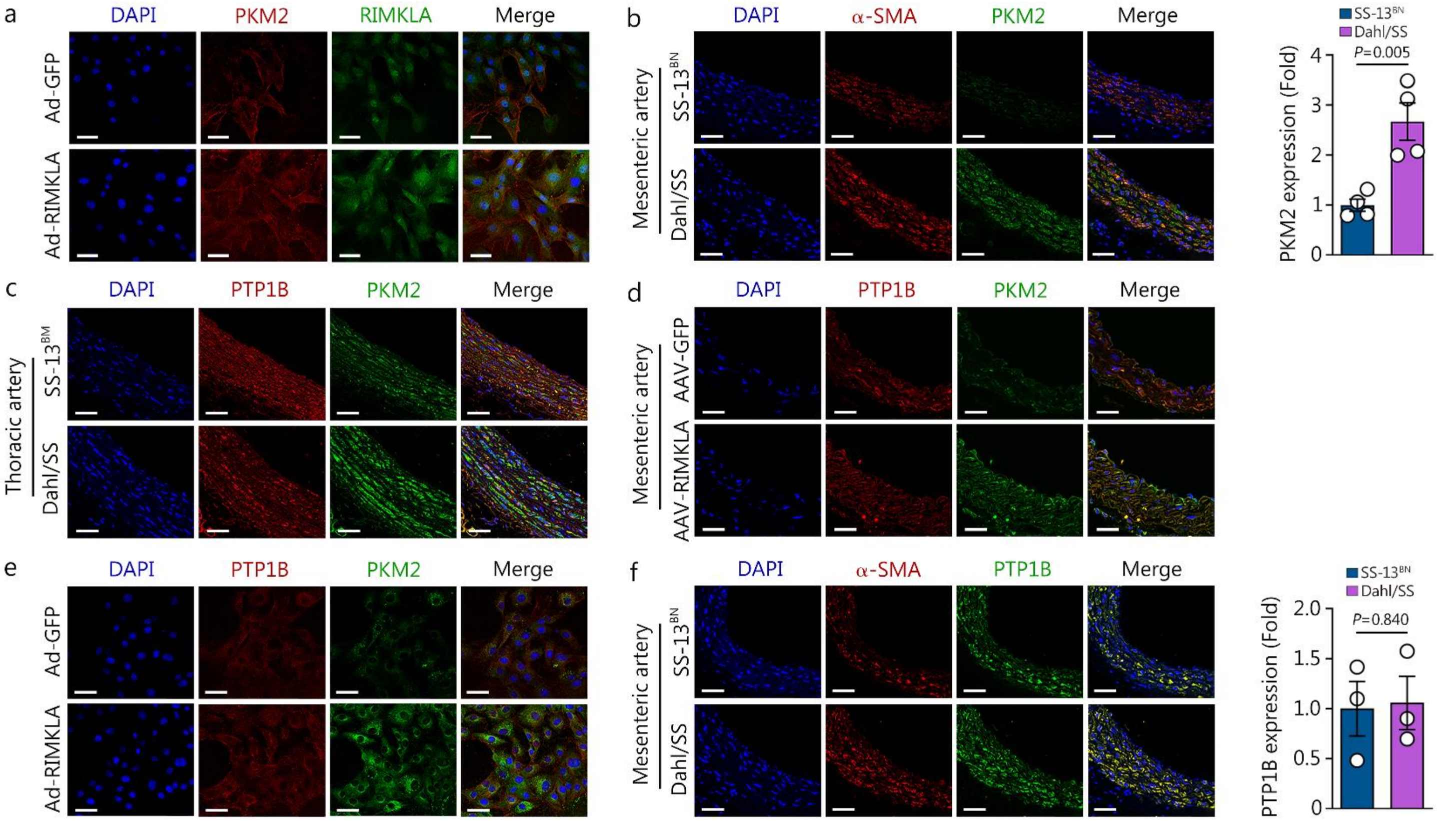

Fig. S12 Colocalizations between RIMKLA and PKM2, as well as PKM2 and PTP1B in VSMCs and arteries. **a** Representative co-staining image of RIMKLA and PKM2 in VSMCs. Scale bar=50 μm. **b** In VSMCs of mesenteric arteries of salt-sensitive hypertensive Dahl/SS rats, PKM2 expression was increased (*n*=4). Scale bar=50 μm. **c** In VSMCs of thoracic arteries of salt-sensitive hypertensive Dahl/SS rats, the colocalization between PTP1B and PKM2 was increased when compared with control rats. Scale bar=50 μm. **d** In VSMCs of the mesenteric artery in mice injected with AAV-RIMKLA, the colocalization between PTP1B and PKM2 was increased when compared with the control mice injected with AAV-GFP. Scale bar=25 μm. **e** Overexpression of *RIMKLA* increased the colocalization between PTP1B and PKM2 in cultured VSMCs. Scale bar=50 μm. **f** In VSMCs of mesenteric arteries of salt-sensitive hypertensive Dahl/SS rats, PTP1B expression exhibited no change when compared with the control rats (*n*=3). Scale bar=50 μm. All *P*-values are analyzed by unpaired *t*-test. VSMC. Vascular smooth muscle cell; RIMKLA. Ribosomal modification protein rimK-like family member A; Ad-RIMKLA. Adenoviral RIMKLA; GFP. Green fluorescent protein; PKM2. Pyruvate kinase M2; PTP1B. Protein-tyrosine phosphatase 1B; DAPI. 4,6'-diamidino-2-phenylindole; α-SMA. α-smooth muscle actin

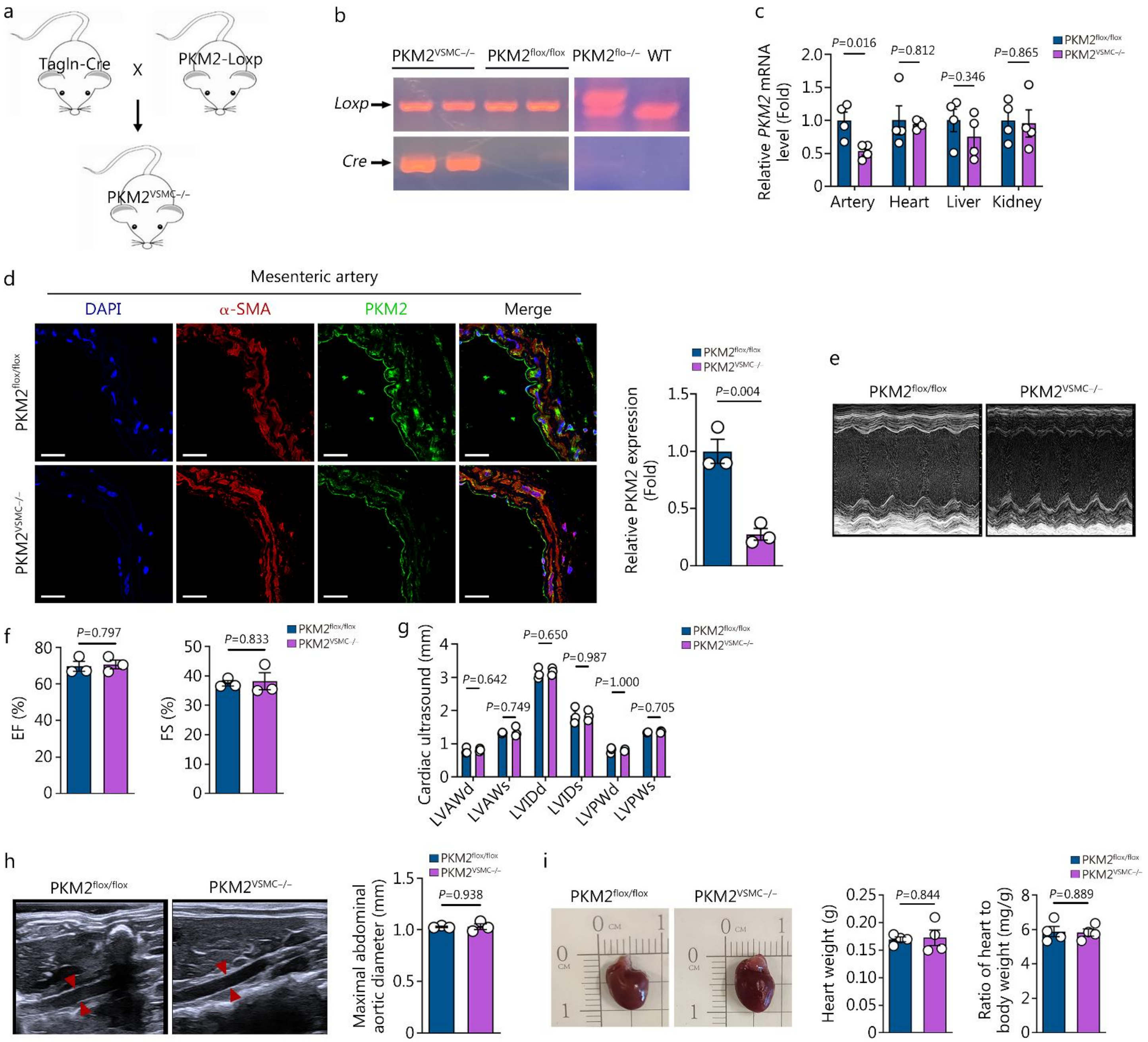

Fig. S13 Generation and characterization of VSMC-specific deletion of *PKM2* in mice. **a** PKM2-Loxp mice were crossed with Tagln-Cre mice to generate PKM2$^{VSMC-/-}$ mice. **b** DNA agarose gel electrophoresis revealed that both *Loxp* and *Cre* genes were expressed in PKM2$^{VSMC-/-}$ mice, while PKM2$^{flox/flox}$ mice only expressed *Loxp* gene. **c** *PKM2* gene mRNA level was reduced in mensenteric artery, but not in heart, liver, and kidney of PKM2$^{VSMC-/-}$ mice (*n*=4). **d** Immunofluorescence staining exhibited that PKM2 expression in VSMCs of mesenteric artery of PKM2$^{VSMC-/-}$ mice was decreased than PKM2$^{flox/flox}$ mice (*n*=3). Scale bar=25 μm. **e** Representative images of cardiac ultrasonography in the PKM2$^{VSMC-/-}$ mice and PKM2$^{flox/flox}$ mice. **f** PKM2$^{VSMC-/-}$ mice had comparable left ventricular ejection fraction (EF) and fractional shortening

(FS) with $PKM2^{flox/flox}$ mice (*n*=3). **g** Quantitative data of cardiac ultrasonography analysis (*n*=3). **h** Representative echocardiographic images of abdominal aorta and quantitative data (*n*=3). **i** Cardiac morphology, heart weight and heart to body weight ratio exhibited no differences between $PKM2^{VSMC-/-}$ mice (8–10-week-old) and $PKM2^{flox/flox}$ mice in physiological condition (*n*=4). All *P*-values are determined by unpaired *t*-test. VSMC. Vascular smooth muscle cell; PKM2. Pyruvate kinase M2; WT. Wild type; DAPI. 4,6'-diamidino-2-phenylindole; α-SMA. α-smooth muscle actin; LVAWd. Left ventricular anterior wall thickness at end-diastole; LVAWs. Left ventricular anterior wall thickness at end-systole; LVIDd. Left ventricular internal dimension at end-diastole; LVIDs. Left ventricular internal dimension at end-systole; LVPWd. Left ventricular posterior wall thickness at end-diastole; LVPWs. Left ventricular posterior wall thickness at end-systole

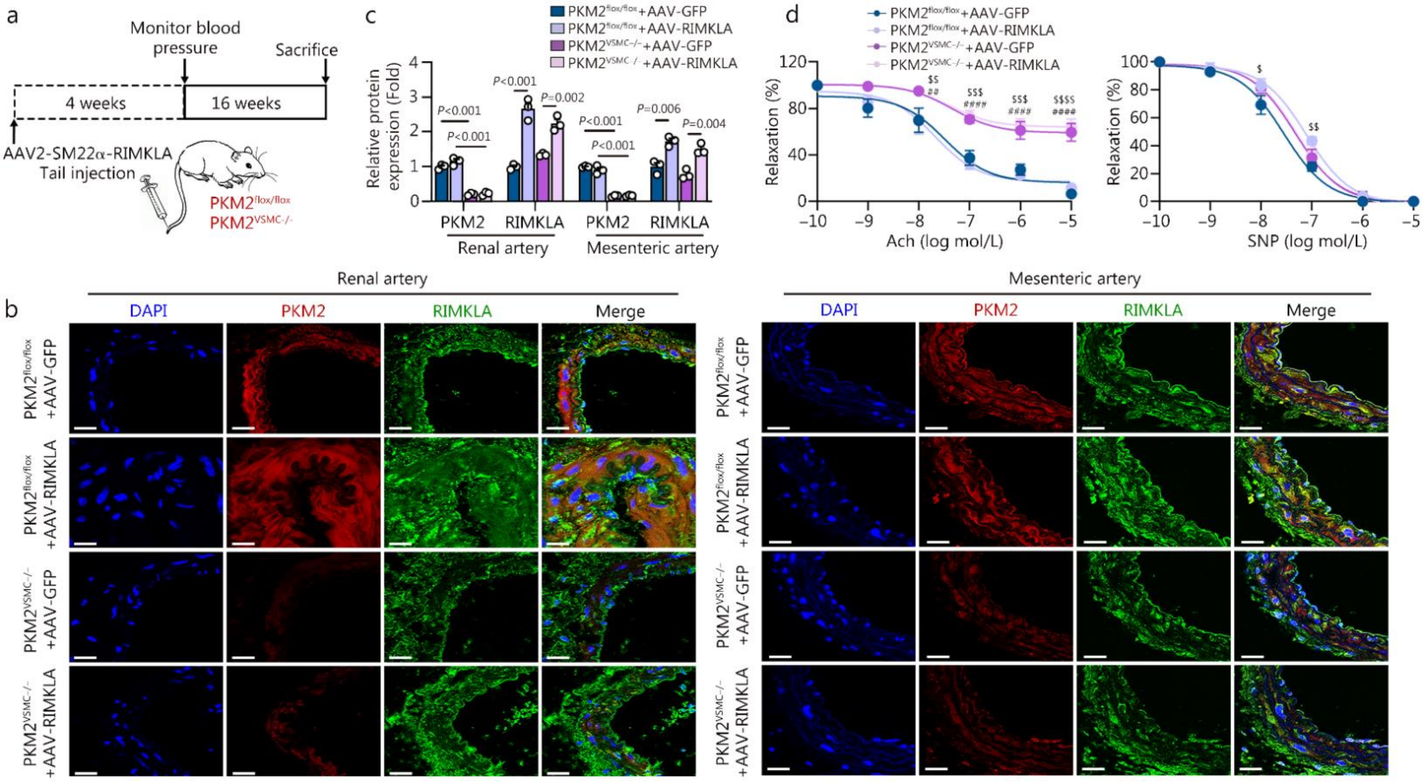

Fig. S14 VSMC-specific overexpression of *RIMKLA* in PKM2$^{VSMC-/-}$ mice. **a** The schematic procedure for VSMC-specific overexpression of *RIMKLA* in PKM2$^{VSMC-/-}$ mice. AAV-RIMKLA was injected via the tail vein to overexpress *RIMKLA* in VSMCs of PKM2$^{VSMC-/-}$ mice. After injection with AAV-GFP/AAV-RIMKLA for 1 month, the blood pressure levels were measured by tail-cuff methods. **b** Immunofluorescent staining of RIMKLA and PKM2 expressions in renal artery and mesenteric artery confirmed the successful conduction of VSMC-specific overexpression of *RIMKLA* in PKM2$^{VSMC-/-}$ mice. Scale bar=25 μm. Quantitative data were presented in panel **c** (*n*=3). **d** The thoracic arteries of PKM2$^{VSMC-/-}$ mice had impaired endothelial-dependent relaxation compared with those of PKM2$^{flox/flox}$ mice (*n*=4). Overexpression of *RIMKLA* failed to affect the endothelial-dependent relaxation of thoracic arteries in PKM2$^{VSMC-/-}$ mice compared with PKM2$^{flox/flox}$ mice. Moreover, overexpression of *RIMKLA* exacerbated endothelial-independent relaxation in PKM2$^{flox/flox}$ mice, with little effect on PKM2$^{VSMC-/-}$ mice. "$" represents PKM2$^{VSMC-/-}$+AAV-GFP vs. PKM2$^{flox/flox}$+AAV-GFP in panel (**d**-left); PKM2$^{flox/flox}$+AAV-RIMKLA vs. PKM2$^{flox/flox}$+AAV-GFP in panel (**d**-right). "#" represents PKM2$^{VSMC-/-}$+AAV-RIMKLA vs. PKM2$^{flox/flox}$+AAV-RIMKLA. $^{\$;\ \#}P<0.05$; $^{\$\$;\ \#\#}P<0.01$; $^{\$\$\$;\ \#\#\#}P<0.001$; $^{\$\$\$\$;\ \#\#\#\#}P<0.0001$. *P*-values are determined by two-way ANOVA corrected using Bonferroni multiple test correction and within-test corrected by Tukey's multiple analysis. VSMC. Vascular smooth muscle cell; AAV. Adeno-associated virus vector; RIMKLA. Ribosomal modification protein rimK-like family member A; PKM2. Pyruvate kinase M2; GFP. Green fluorescent protein; DAPI. 4,6'-diamidino-2-phenylindole

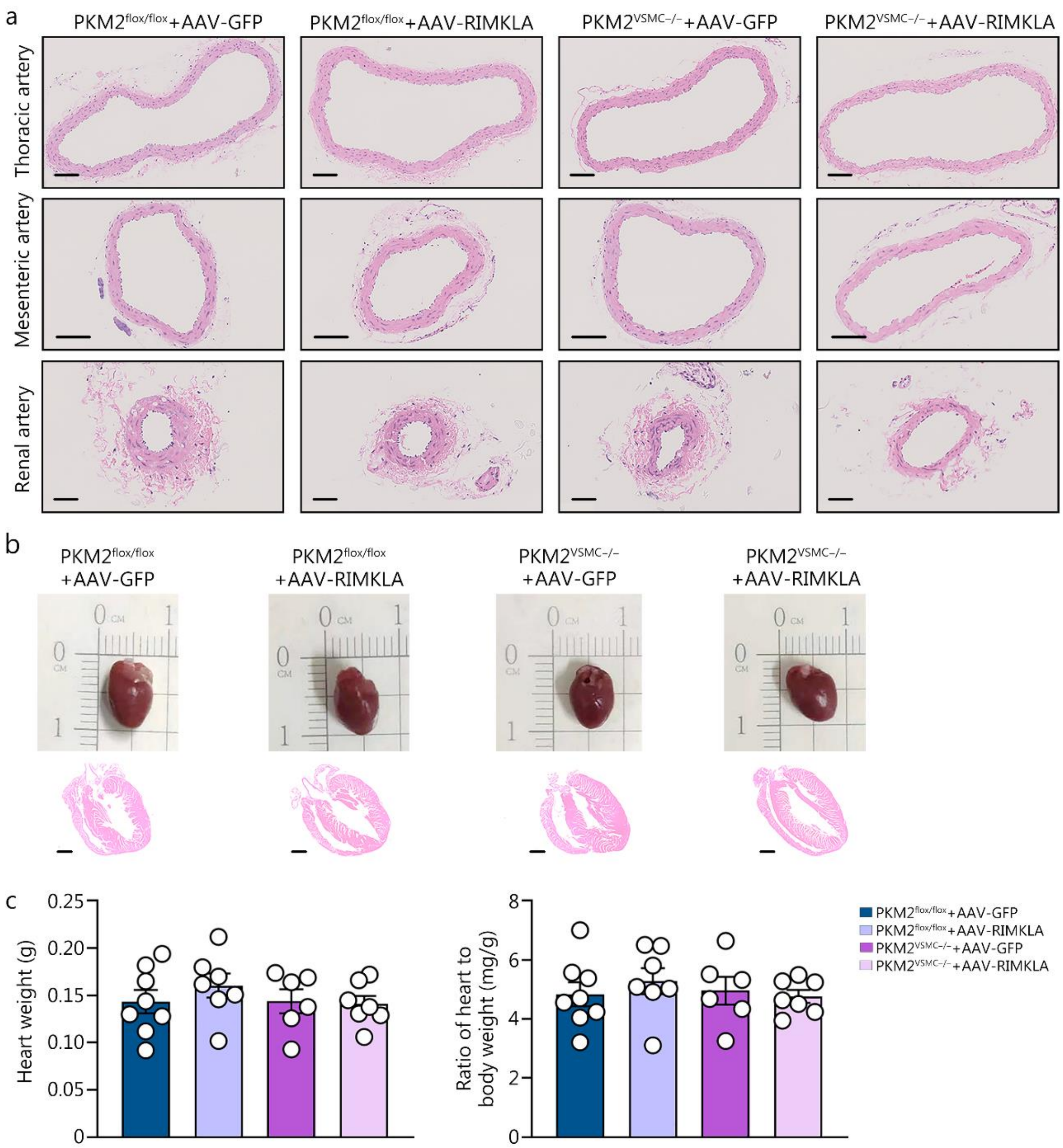

Fig. S15 VSMC-specific overexpression of *RIMKLA* has little effect on cardiovascular remodeling in PKM2$^{VSMC-/-}$ mice. **a** H&E staining of the thoracic artery, mesenteric artery, and renal artery revealed no vascular remodeling in PKM2$^{VSMC-/-}$ mice with or without *RIMKLA* overexpression. Scale bar=50 μm for the last row of images, 100 μm for others. Cardiac morphology (**b**), heart weight and heart to body weight ratio (**c**) exhibited no differences between PKM2$^{VSMC-/-}$ mice and PKM2$^{flox/flox}$ mice after injection with AAV-GFP or AAV-RIMKLA (*n*=6–7). Scale bar=1 mm. *P*-values are determined by one-way ANOVA corrected using Bonferroni multiple test and within-test corrected by Tukey's multiple analysis. VSMC. Vascular smooth muscle cell; AAV. Adeno-associated virus vector; RIMKLA. Ribosomal modification protein rimK-like family member A; PKM2. Pyruvate kinase M2; GFP. Green fluorescent protein

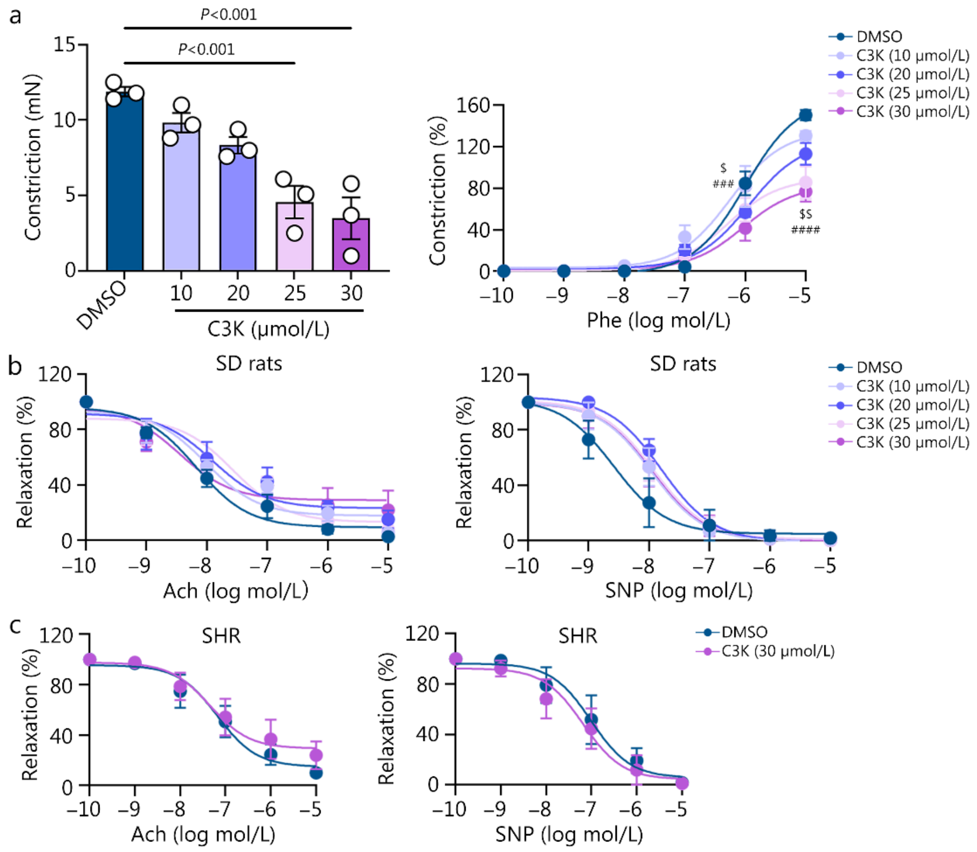

Fig. S16 Treatment with PKM2 inhibitor dose-dependently reduces arterial vasoconstriction of SD rats. Superior mesenteric arteries of SD/SHR rats were preincubated in various concentrations of PKM2 inhibitor C3K for 6 h before constriction and relaxation were determined. C3K dose-dependently inhibited potassium and Phe-induced constriction of arteries (**a**) with little effect on Ach- and SNP-induced relaxation of SD arteries (**b**) (*n*=3). $^{\$}P<0.05$, $^{\$\$}P<0.01$, 20 μmol/L C3K vs. DMSO; $^{\#\#\#}P<0.001$, $^{\#\#\#\#}P<0.0001$, 30 μmol/L C3K vs. DMSO. **c** Treatment with 30 μmol/L C3K had little effect on endothelial-dependent (Ach-induced) and endothelial-independent (SNP-induced) relaxation of SHR arteries (*n*=5). *P*-value for (a) is determined by one-way ANOVA corrected using Bonferroni multiple test, and others are analyzed by two-way ANOVA corrected using Bonferroni multiple test and within-test corrected by Tukey's multiple analysis. PKM2. Pyruvate kinase M2; SD. Sprague-Dawley; SHR. Spontaneously hypertensive rat; Phe. Phenylephrine; Ach. Acetylcholine; SNP. Sodium nitroprusside dehydrate

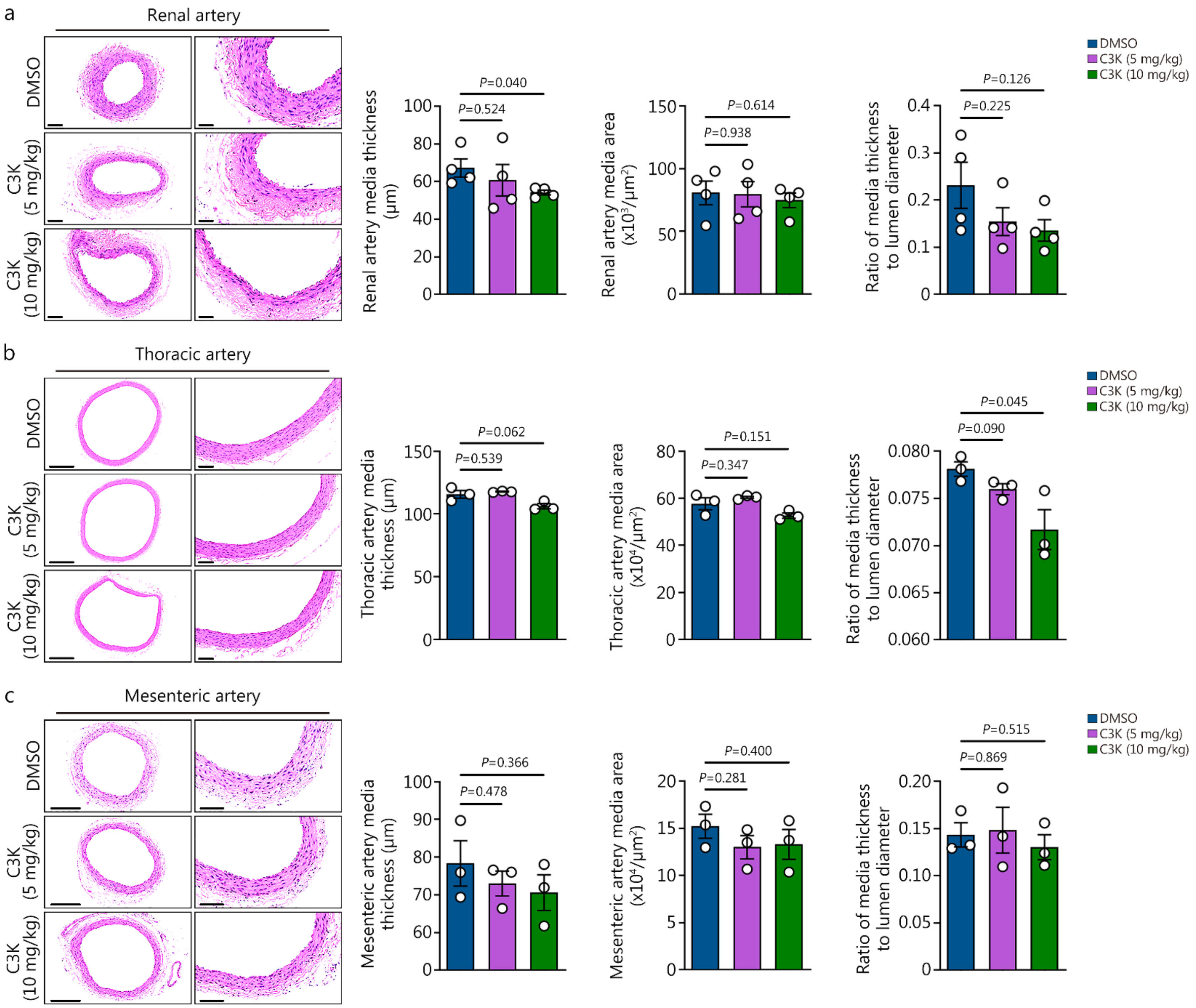

Fig. S17 Administration with PKM2 inhibitor alleviates artery remodeling of SHR. **a** Representative H&E staining images of renal artery of SHR after treating with PKM2 inhibitor [C3K 5 mg/(kg d); 10 mg/(kg d)] for 1 week. Scale bar=100 μm (left) or 50 μm (right). Quantitative data analysis of renal media thickness, media area, and ratio of media thickness to lumen diameter (*n*=4). **b** Representative H&E staining images of thoracic artery of SHR after treating with PKM2 inhibitor. Scale bar=500 μm (left) or 100 μm (right). Quantitative data analysis of thoracic media thickness, media area, and ratio of media thickness to lumen diameter (*n*=3). **c** Representative H&E staining images of mesenteric artery of SHR after treating with PKM2 inhibitor. Scale bar=250 μm (left) or 100 μm (right). Quantitative data analysis of mesenteric media thickness, media area, and ratio of media thickness to lumen diameter (*n*=3). All *P*-values are determined by unpaired Student's *t*-test. PKM2. Pyruvate kinase M2; SHR. Spontaneously hypertensive rat

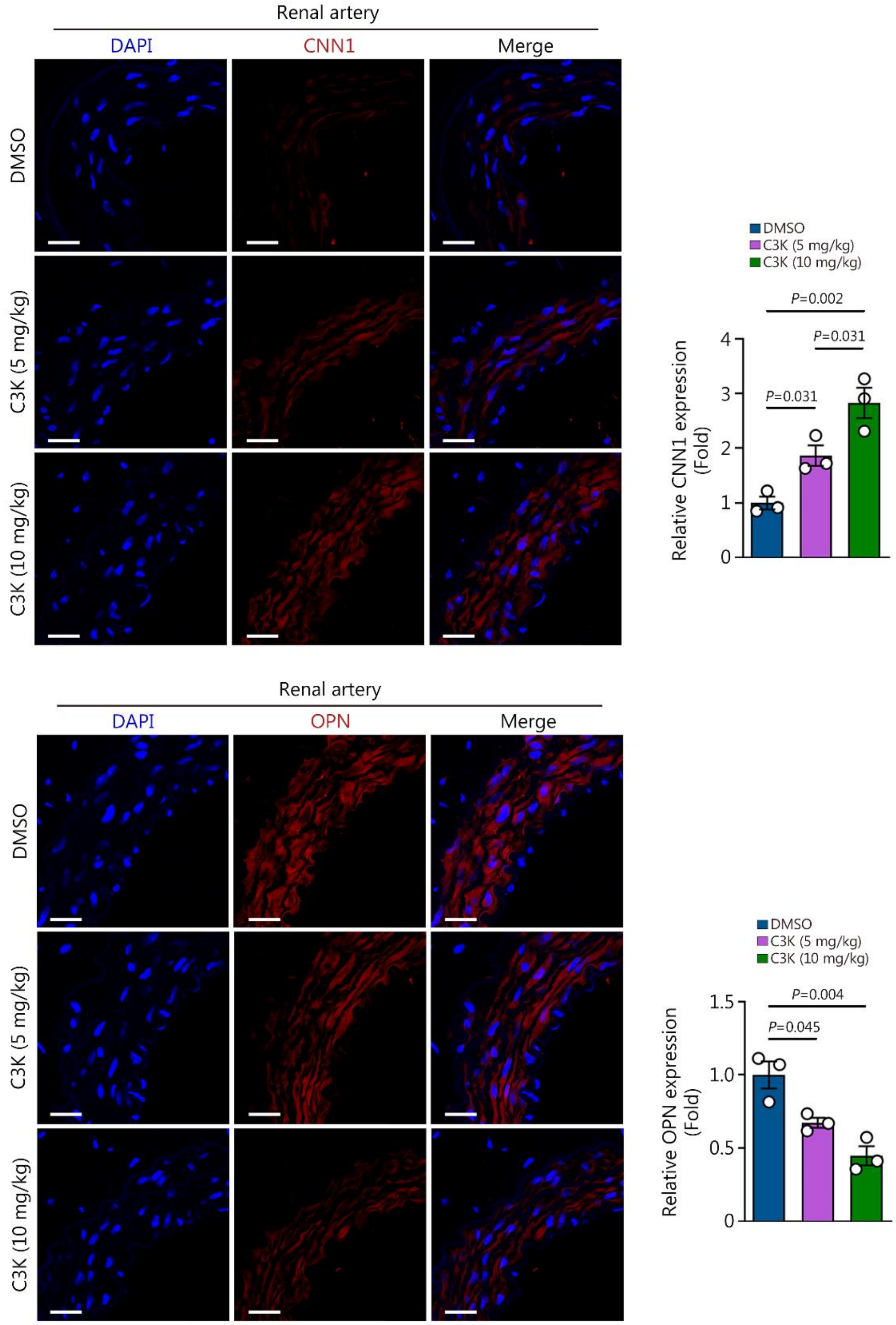

Fig. S18 PKM2 inhibitor dose-dependently reversed VSMC phenotype switch in renal arteries of SHR. Representative immunofluorescent staining images of CNN1 protein and OPN protein in the renal artery of SHR after treating with PKM2 inhibitor [C3K 5 mg/(kg d); 10 mg/(kg d)] for 1-week (*n*=3). Scale bar=25 μm. *P*-values are determined by one-way ANOVA followed by Bonferroni post hoc analysis. PKM2. Pyruvate kinase M2; SHR. Spontaneously hypertensive rat; VSMC. Vascular smooth muscle cell; OPN. Osteopontin; CNN1. Calponin 1

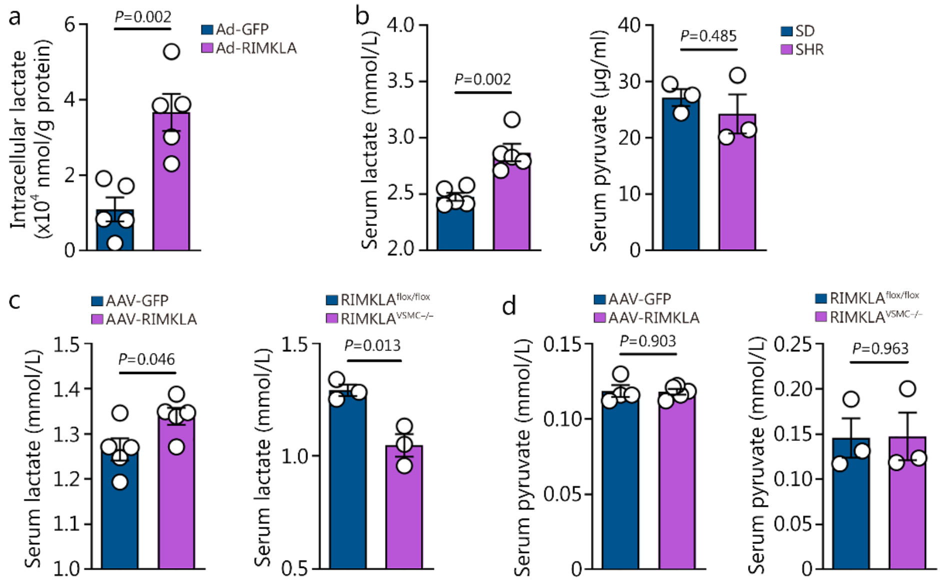

Fig. S19 Overexpression of *RIMKLA in vitro* and *in vivo* increased lactate content. **a** *RIMKLA* overexpression enhanced lactate production in VSMCs (*n*=5). **b** Serum lactate rather than pyruvate was increased in SHR compared to SD rats (*n*=3–5). **c** Serum lactate was increased in AAV-RIMKLA mice, and decreased in RIMLA$^{VSMC-/-}$ mice compared with control mice (*n*=3–5). **d** VSMC-specific overexpression or deletion of *RIMKLA* resulted in comparable serum pyruvate compared to control mice (*n*=3–5). All *P*-values are determined by unpaired *t*-test. VSMC. Vascular smooth muscle cell; RIMKLA. Ribosomal modification protein rimK-like family member A; Ad-RIMKLA. Adenoviral RIMKLA; GFP. Green fluorescent protein

References

1. Xiang R, Chen J, Li S, Yan H, Meng Y, Cai J*, et al.* VSMC-specific deletion of *FAM3A* attenuated Ang II-promoted hypertension and cardiovascular hypertrophy. Circ Res. 2020;126(12):1746-59. https://doi.org/10.1161/circresaha.119.315558.
2. Yan H, Liu W, Xiang R, Li X, Hou S, Xu L*, et al.* Ribosomal modification protein rimK-like family member A activates betaine-homocysteine S-methyltransferase 1 to ameliorate hepatic steatosis. Signal Transduct Target Ther. 2024;9(1):214. https://doi.org/10.1038/s41392-024-01914-0.
3. Angerani S, Lindberg E, Klena N, Bleck CKE, Aumeier C, Winssinger N. Kinesin-1 activity recorded in living cells with a precipitating dye. Nat Commun. 2021;12(1):1463. https://doi.org/10.1038/s41467-021-21626-1.
